# Recent advances in optical label-free characterization of extracellular vesicles

**DOI:** 10.1515/nanoph-2022-0057

**Published:** 2022-04-13

**Authors:** Meruyert Imanbekova, Sorina Suarasan, Yao Lu, Sarah Jurchuk, Sebastian Wachsmann-Hogiu

**Affiliations:** Bioengineering, McGill University Faculty of Engineering, Montreal, QC, Canada; Nanobiophotonics and Laser Microspectroscopy Center, Interdisciplinary Research Institute in Bio-Nano-Sciences, Babes-Bolyai University, T. Laurian 42, 400271, Cluj-Napoca, Romania; Bioengineering, McGill University Faculty of Engineering, 3480 Rue Universite, 1006, Montreal, QC, H3C6W1, Canada; Bioengineering, McGill University Faculty of Engineering, 3480 Rue Universite, Rm#350, Montreal, QC, H3A 0E9, Canada; Bioengineering, McGill University Faculty of Engineering, 3480 University St., MC362, Montreal, H3A 0E9l, Canada

**Keywords:** early disease diagnosis, extracellular vesicles, label-free detection, liquid biopsy, optical methods

## Abstract

Extracellular vesicles (EVs) are complex biological nanoparticles endogenously secreted by all eukaryotic cells. EVs carry a specific molecular cargo of proteins, lipids, and nucleic acids derived from cells of origin and play a significant role in the physiology and pathology of cells, organs, and organisms. Upon release, they may be found in different body fluids that can be easily accessed *via* noninvasive methodologies. Due to the unique information encoded in their molecular cargo, they may reflect the state of the parent cell and therefore EVs are recognized as a rich source of biomarkers for early diagnostics involving liquid biopsy. However, body fluids contain a mixture of EVs released by different types of healthy and diseased cells, making the detection of the EVs of interest very challenging. Recent research efforts have been focused on the detection and characterization of diagnostically relevant subpopulations of EVs, with emphasis on label-free methods that simplify sample preparation and are free of interfering signals. Therefore, in this paper, we review the recent progress of the label-free optical methods employed for the detection, counting, and morphological and chemical characterization of EVs. We will first briefly discuss the biology and functions of EVs, and then introduce different optical label-free techniques for rapid, precise, and nondestructive characterization of EVs such as nanoparticle tracking analysis, dynamic light scattering, atomic force microscopy, surface plasmon resonance spectroscopy, Raman spectroscopy, and SERS spectroscopy. In the end, we will discuss their applications in the detection of neurodegenerative diseases and cancer and provide an outlook on the future impact and challenges of these technologies to the field of liquid biopsy *via* EVs.

## 1 Introduction

Extracellular vesicles (EVs) are heterogeneous lipid membrane-enclosed, nanometer-sized vesicles shed by all cells in the human body. Their molecular cargo contains lipids, proteins, nucleic acids, and sugars, and may carry biomarkers of certain diseases [1]. The uptake of EVs by recipient cells triggers intercellular signaling and can further activate intracellular metabolic pathways [2]. Determining the role and impact of EVs in a variety of cellular functions and disease states holds potential for improved diagnostics and therapeutics. In this context, it is important to establish quantitative characterization methodologies related to the three aspects of EVs research: morphology, biochemical composition, and functions.

**Table 1:** Summary of label-free optical particle counting, sizing, and morphology characterization methods.

Method	Morphological information	Data acquisition time	Sample type Liquid/dry	Advantages	Disadvantages	References
NTA	Size: 30–1000 nm Size distribution Refractive index Counting	Minutes	Liquid	Size distribution of individual EVs, high throughput	Bias towards overestimation of large particle concentration Requires large sample volumes	[71, 75, 76, 118]
DLS	Size: 1–6000 nm Size distribution	Minutes	Liquid	High throughput analysis of EVs size, concentration and shape	Bias towards large particles Low resolution cannot distinguish small particles from each other	[80, 83, 85]
AFM	Morphology: nm Topography: nm EV density Size Adhesion, deformation, elastic modulus	∼Hour per sample	Air (more stable, faster) Liquid (more accurate size)	Single EV analysis, enables measurements of mechanical properties of EVs with high resolution (lateral resolution 1–3 nm)	Mechanical stress Low throughput Labor intensive	[95, 109, 122, 125]
FC	Size: >300 nm (conventional) 100 nm (high-resolution fluorescent tagging) Counting	Minutes	Liquid	Single EV analysis, determinates concentration, size distribution, and biochemical cargo characterization, high throughput	Variability between instruments and runs Few instruments can detect below 300 nm with the use of fluorescent labels only	[66, 127, 133, 134]
SRM	50–1000 nm	Seconds	Liquid	Single EV analysis, allows investigation of EVs functions *in vivo*/*in situ* with molecular resolution	Requires labels	[142]
SP-IRIS	Single EV size: 400 nm Counting	Minutes	Dry	Single EV analysis, allows simultaneous measurement of EVs size and surface markers	Bias towards large particles in highly concentrated EVs samples	[68, 143, 144]

**Table 2:** Summary of label-free optical methods for EVS biochemical composition characterization.

Method	Biochemical information	Data acquisition time	Sample type Liquid/dry	Advantages	Disadvantages	References
Raman spectroscopy	Protein, lipid, nucleic acids, metabolites, and saccharides	Minutes	Liquid/dry	Quantitative and qualitative characterization of surface and internal biochemical content of EVs, is able to reveal conformational form and structure of proteins and lipids, minimal preprocessing, small, sample size	Weak Raman scattering, Low throughput	[156, 170]
SERS	Protein, lipid, nucleic acids, metabolites, and saccharides	Minutes	Liquid/dry	Quantitative and qualitative characterization of surface biochemical content of EVs, is able to reveal conformational form and structure of proteins and lipids, minimal preprocessing, small sample size	Limited by the distance between biomolecule and the SERS substrate, suitable for characterization of membrane bounded molecules	[211, 212]
SPR	Specific molecules (proteins and lipids) of interest	∼Hour	Liquid	Real-time monitoring of EVs-ligand binding kinetics, small sample size	Labor intensive, limited by use of capturing molecules	[190]
IR spectroscopy	Protein, lipid, nucleic acids, and saccharides	Seconds	Liquid/dry	Quantitative and qualitative characterization of EVs biochemical content, minimal preprocessing, small sample size	Low throughput	[198]
Multiphoton microscopy	Metabolites	Seconds	Liquid/dry	Single EV analysis, allows investigation of EVs functions *in vivo*/*in situ*	Limited by penetration depth (250–500 μm), costly, phototoxicity	[213]

While EVs exhibit significant heterogeneity, establishing distinct subpopulations of EVs has been a persistent problem in the EVs research field. EVs are known to vary in size, biogenesis pathways, cells of origin, morphology, molecular cargo, and functions [1, 3]. EVs range in size from 50 to 2000 nm and are commonly divided into three main types based on their biogenesis pathway: exosomes, microvesicles, and apoptotic bodies. These will be discussed more in-depth later in this review. Briefly, exosomes or small extracellular vesicles have an endosomal origin and are typically less than 150 nm, microvesicles formed by outward budding and fission of cell plasma membrane and their size ranges between 50 nm and 2000 nm, and apoptotic bodies are a heterogeneous population of EVs formed during apoptosis [2], [3], [4]. However, due to overlapping size ranges, current EVs isolation methods are not able to precisely differentiate between subtypes of EVs. In addition, resulting EVs pellets may contain other co-isolated particles present in the cell culture media or biofluids such as viruses and protein aggregates. Therefore, to uncover EVs heterogeneity, a comprehensive assessment of their morphology and biochemical composition is needed.

**Figure 1:**
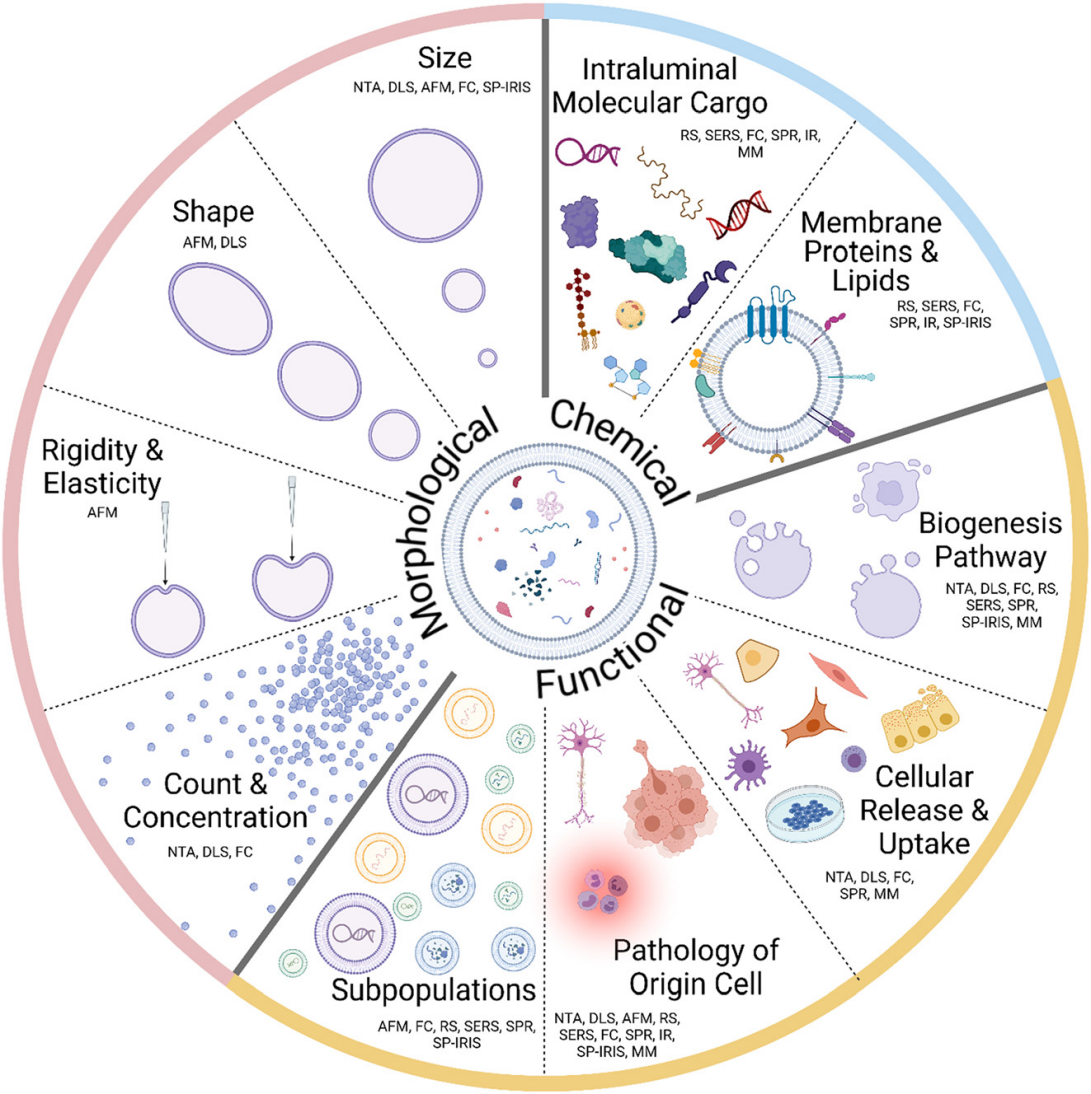
Optical label-free characterization methodologies for EV heterogeneity characterization. Categories of EVs heterogeneity are divided into morphological, biochemical, and functional heterogeneity. *Morphological heterogeneity* category includes size, shape, rigidity, elasticity, count, and concentration. Size of individual EVs can be determined by NTA, DLS, AFM, FC, and SP-IRIS. The size distributions within a population of EVs, as well as the count and concentration of EVs can be determined with NTA, DLS, and FC. The shape of EVs can be characterized using AFM and DLS. EVs rigidity and elasticity can only be determined by AFM. *Biochemical heterogeneity* of EVs is derived from intraluminal molecular cargo and membrane molecules. RS, SERS, FC, SPR, IR-FTIR, SP-IRIS, and MM are the methods that are applied to characterize EVs biochemical content. *Functional heterogeneity* of EVs derives from the state of the cell of origin, biogenesis pathway and heterogeneity of EVs within subpopulations (exosomes, microvesicles, and apoptotic bodies). EVs biogenesis pathway is commonly determined by combining size and biochemical content characterization methods including NTA, DLS, FC, RS, SERS, SPR, SP-IRIS, and MM. The cells of EVs origin as well as their physiological state are determined by analyzing EVs morphological features and their biochemical content, where the differences in EVs counts, shape, rigidity, and elasticity can indicate disease triggered alterations in the cell of origin. NTA, DLS, AFM, RS, SERS, FC, SPR, IR-FTIR, SP-IRIS, and MM are applied to study the pathological functions of EVs. The dynamics of EVs release and uptake may be characterized *via* NTA, DLS, FC, MM, and SPR. Finally, EVs are found to vary in size, morphology, and biochemical cargo even within one specific subpopulation. These differences are currently studied using AFM, RS, SERS, SPR, FC, and SP-IRIS.

The morphological heterogeneity of EVs is mainly described by their differences in size, shape, and elasticity (Figure 1). The wide range in size, from 50–2000 nm, offers a particularly unique window for exploration. In terms of shape, exosomes and microvesicles are generally spherical or ellipsoid shapes [1], while apoptotic bodies have heterogeneous morphologies and are present in a wide variety of shapes and sizes [5].

The variations in EVs’ biochemical composition are ultimately the result of their different biogenesis and subsequent cargo loading [6] and reflect the state of releasing cells [7, 8]. Specifically, the molecular cargo of EVs includes proteins [9], lipids [10], various subpopulations of RNA [11], and DNA [12]. Hence, an accurate assessment of EVs’ biochemical composition is important for anticipation of their potential functions in normal physiology as well as to uncover their relevance to disease.

Given the vast heterogeneity of EVs discussed before, there are extensive challenges in their isolation and characterization. No single characterization technique or isolation method can capture the full-size range of EVs. Some morphological characterization and counting techniques have also been shown to be biased towards certain size ranges, due to technical limitations and calibration issues [13, 14]. EVs samples are rarely fully purified or isolated [14]. The overlap in size and chemical composition makes the separation of specific EVs subpopulations challenging. In addition, the variations in protein, lipid, and nucleic acid profiles mean that labelling and affinity methods can only capture some information within limited populations of EVs. Therefore, combinations of methods are often used to extract EVs and further explore their biochemical composition [13]. This leads to the need of standard methodologies for quantitative EVs characterization. However, the repeatability of results is a common weakness within the field. Therefore, the International Society for Extracellular Vesicles (ISEV) has established guidelines for the Minimal Information for Studies of Extracellular Vesicles, most recently updated in 2018 (MISEV2018) [14]. The guidelines outline the need for specific EVs terminology as well as detailed reporting on the isolation and EVs characterization techniques used.

Current methodologies of EVs characterization may employ labels or can be label-free. A large variety of labeling and nonlabeling characterization techniques have been developed to address the processing and testing needs of different EVs subgroups [15].

The field of label-based techniques is rapidly expanding with advancements in high-resolution microscopy techniques as well as improvements in EVs labeling strategies. Labeling involves the use of molecular tags or markers that bind to EVs. Label types can include fluorescent dyes and molecules [16], radionuclides [17], and lipophilic tracer dyes [18]. The presence of the label is measured downstream and can be used to characterize the EVs. Label-based methods offer a range of advantages such as simple differentiation, visualization, and tracking of different EVs. They can be implemented into high-throughput strategies, such as flow cytometry. Labeling is also applied for experimental validation when combined with techniques for single-molecule characterization, such as high-resolution microscopy [19–21]. Moreover, labels are also highly practical for acting as positive or negative markers to ensure adequate measurement and characterization of individual target particles [22]. Despite these useful functions that labels can fulfill, there are some fundamental concerns that may arise when using label-based methods. First, because of the vast heterogeneity of the surface proteins and molecular content of EVs, there is no established single optimal positive or negative marker for EVs recognition or characterization [14] that can be used in label-based methods. Furthermore, one of the main drawbacks of label-based techniques is the potential unwanted and unanticipated interaction of labels with EVs which can compromise the obtained data and lead to false conclusions. Moreover, EVs’ functions or their interaction with cells can be impaired or obstructed by the label itself, leading to misleading data on EVs uptake [23]. Another potential problem is the aggregation of labels or consequent labeling of non-EVs particles that may cause false-positive results [24, 25]. Finally, labels may also degrade or change over time including photobleaching of fluorescent tags [26], or end up as hazardous waste, such as radiolabels. These limitations of the labelling methodologies necessitate the use of label-free methods for certain applications.

Label-free techniques are a variety of methods that do not use tags or labels to detect and characterize the analyte of interest. These methods are often based on direct and noninvasive probing of the inherent features of the analyte. Elimination of the need for tags or dyes results in numerous advantages. First, the lack of a tag allows performing measurements in conditions close to physiological which are beneficial for both fundamental and applied research. For example, identification, quantification, and characterization of proteins in native conformation by label-free mass spectrometry and Raman spectroscopy. Furthermore, label-free techniques require less time and wet-lab complexity compared to labelling techniques which consequently leads to fewer wet lab errors. In the field of EVs research, label-free methods offer a non-invasive approach for studying EVs molecular cargo including proteins conformation, and lipids structures that may further affect EVs functions. However, these technologies have some challenges and limitations that will be extensively discussed in this review.

Among label-free techniques, optical methods that utilize light and optical properties for noninvasive analysis of EVs size, morphology, concentration, and molecular content are particularly interesting. Raman spectroscopy, surface enhanced Raman spectroscopy (SERS), and Fourier-transform infrared (FTIR) spectroscopy are examples of optical methods that have been used for characterization of EVs biochemical content, while techniques such as nanoparticle tracking analysis (NTA), atomic force microscopy (AFM), and dynamic light scattering (DLS) are often applied to reveal morphological features of EVs. In addition, there are novel emerging optical single-vesicle characterization approaches such as Raman tweezers microspectroscopy, AFM coupled infrared (IR) spectroscopy, and single particle interferometric reflectance imaging sensor (SP-IRIS) that are capable to reveal specific features of a single vesicle and shine light on EVs heterogeneity.

In this review, we summarize current optical label-free methods used to study EVs and their contributions to understanding EVs biology and EVs associated pathology of various diseases. We will first elaborate on EVs’ biology and functions (Section 2). This section will break down the main classifications of EVs into exosomes, microvesicles, and apoptotic bodies, with a focus on their biogenesis pathways, molecular cargo, and their functions. Next, we will discuss morphological characterization, counting, and sorting methods such as NTA, AFM, flow cytometry (FC), DLS, and SP-IRIS (Section 3). This section will first explain their working principles and key characteristics, and then we will discuss main advantages and disadvantages of each method. In Section 4 we will describe optical technologies that are applied for EVs molecular content characterization including Raman spectroscopy, SERS, surface plasmon resonance (SPR) spectroscopy, IR spectroscopy, and multiphoton microscopy (MM). Subsequently, in Section 5 we will define application of the aforementioned techniques for exploration of pathophysiology and diagnostics of various diseases. Finally, Section 6 will overview our perspectives and conclusions on label-free optical characterization techniques and applications within the rapidly growing field of EVs research.

## 2 Biology and functions of EVs

The current state of knowledge identifies EVs as small lipid-membrane enclosed heterogeneous structures. There are a number of review articles that describe in great detail the aspects of EVs biology [27, 28]. For the purpose of this review, we will briefly outline EVs subtypes and their main characteristics. Their size ranges from 50 nm to 2000 nm in diameter depending on a subtype of EVs. The three major subtypes of EVs based mainly on their biogenesis are exosomes (less than 150 nm in diameter), microvesicles, and apoptotic bodies (both considered to be larger than 200 nm) [29]. Some studies classify EVs according to their origin (ectosomes, prostasomes, cardiosomes, and mitovesicles). All EVs subtypes share important characteristics such as lipid bilayer membrane, ability to carry intraluminal cargo of proteins and nucleic acids, and their release into extracellular space [2]. Although the amount of research aimed to study the biology of EVs has increased over the past few decades, the detailed mechanisms of their biogenesis, cargo loading, trafficking, and release are not completely understood.

### 2.1 Exosomes

**Figure 2:**
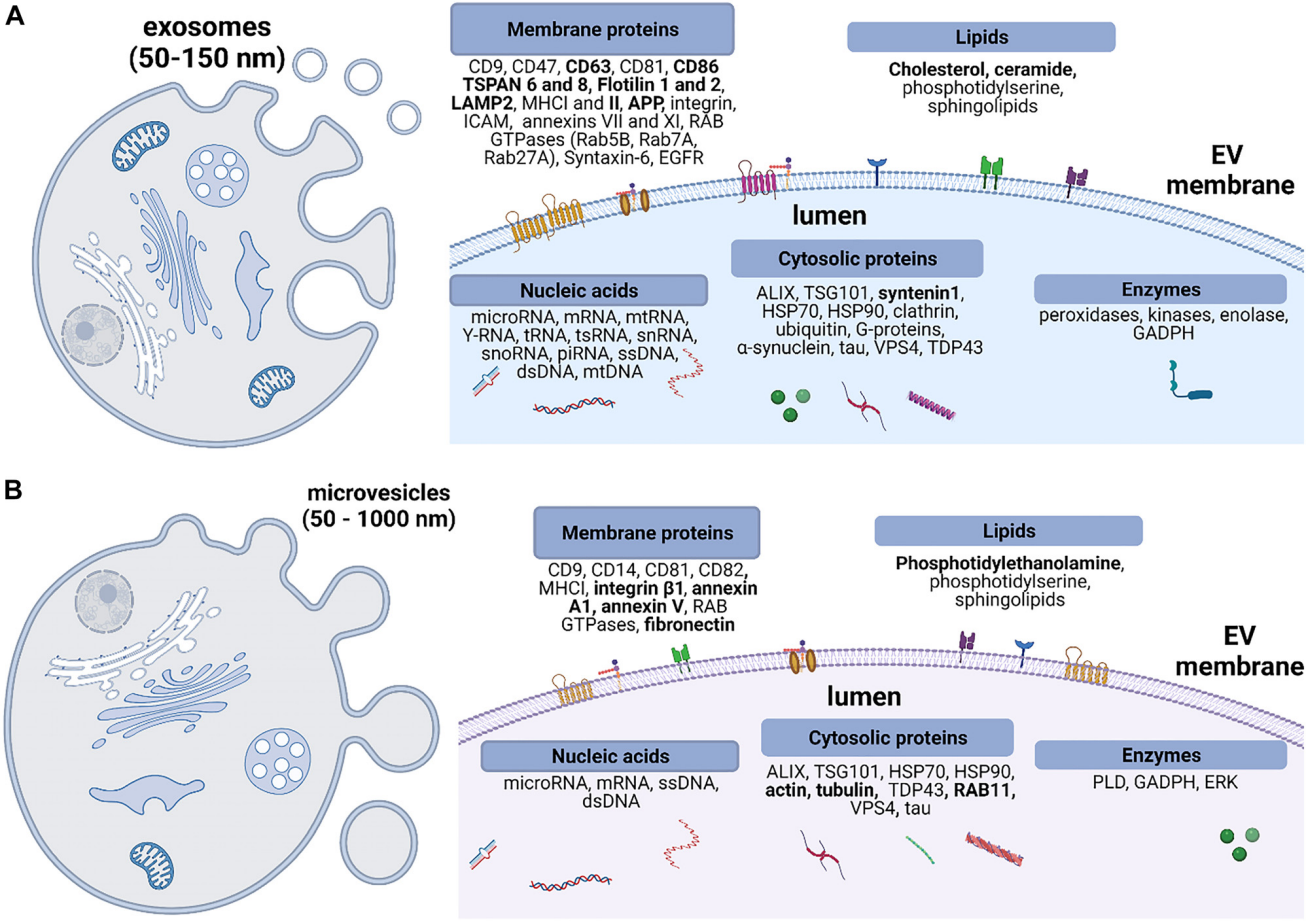
Biogenesis and chemical composition of a single EV. (A) Exosomes are released upon fusion of multivesicular bodies (MVBs) to an extracellular matrix. Exosomes have a phospholipid bilayer membrane and carry various species of proteins, nucleic acids, and metabolites. (B) Microvesicles form *via* outward budding of the cell plasma membrane. Their biochemical content has some similarities with exosomes’ and includes a variety of membrane proteins, cytosolic proteins, lipids, and nucleic acids. Bold fonts in both panels represent EVs molecular cargo that is uniquely found or enriched either in exosomes or in microvesicles. TSPAN, tetraspanins; APP, amyloid precursor protein; LAMP2, lysosomal associated membrane protein 2; MHC, major histocompatibility complex; ICAM, intercellular adhesion molecule; EGFR, epidermal growth factor receptor; ALIX, ALG-2 interacting protein; TSG101, tumor susceptibility gene 101 protein; HSP, heat shock protein; VPS, vacuolar soring associated protein; TDP43, TAR DNA-binding protein 43; GADPH, glyceraldehyde-3-phosphate dehydrogenase; PLD, phospholipase D; ERK, extracellular receptor kinase.

Exosomes, or small extracellular vesicles (sEVs), are membrane-bound EVs that originate from an endosomal pathway and are proposed to carry intercellular cargo for cell-to-cell communication as seen in Figure 2A [29]. Exosomes are released into the extracellular space upon fusion of the MVBs or late endosomes with the plasma membrane [2]. This tightly regulated process of intraluminal vesicles (ILVs) formation and cargo loading depends on the endosomal sorting complex required for transport (ESCRT) machinery (ESCRT-0, -I, -II, -III) [30, 31]. The initial step in this process is the formation of an early endosome (EE) by fusion of the primary endocytic vesicles containing receptors and proteins, which are regulated by clathrin- or caveolin mediated formation of inward budding of the plasma membrane [32]. Rab 5 protein plays a crucial role in the early stages of EE formation and the conversion to late endosome or MVB [33]. Next step is the formation of ILVs in late endosomes. ESCRT-0 and ESCRT-I cluster ubiquitinated proteins on the MVBs membrane and generate inward budding and scission of the limiting membrane *via* ESCRT-II and ESCRT-III subunits [34] as well as syntenin, and ALG-2 interacting protein X (ALIX) [35].

Additionally, there is another possible way of exosome formation known as the ESCRT-independent mechanism that involves ceramide [36, 37] and tetraspanins [38]. Ceramide forms membrane subdomains that can be spontaneously budded inside the MVB [37]. In addition, proteins of the tetraspanin family such as CD63, CD9, CD81, and CD82 are involved in exosome formation and cargo sorting [38]. These proteins are suggested to have a cone-shaped structure that contains cholesterol molecules and their enrichment in specific microdomains can induce inward budding of the membrane [39]. Both described pathways seem to be active in exosome formation and their contribution may be affected by the cell type. Little is known about the molecular mechanisms that regulate the fate of MVBs after their formation. The MVBs may be degraded by a lysosome or fuse with the plasma membrane which further leads to exosome release. The exosome biogenesis pathway requires transport of MVBs to the plasma membrane. This step involves association of MVBs with cytoskeleton components such as actin and microtubules, molecular motors, and molecular switches (Rab family proteins) [34]. Rab 7 and dynein are further promoted to transport MVBs toward the plasma membrane [40]. Another protein of the Rab family, Rab 27, is found to regulate fusion of MVBs with plasma membrane by rearranging actin cytoskeleton [41]. It has been shown that this process is determined by soluble factors such as N-ethylmaleimide-sensitive factor (NSF) and soluble NSF-attachment protein (SNAP) as well as membrane complexes such as SNAP-attachment protein receptor (SNARE). The release of exosomes into the extracellular matrix may be regulated by Ca^2+^ and is based on ATP-dependent interaction of actin and myosin leading to cytoskeleton contraction [42, 43].

Subsequently, the molecular cargo of exosomes includes a large variety of proteins that are involved in their biogenesis, such as membrane proteins including tetraspanins (CD9, CD63, CD81, CD86, TSPAN 6 and 8, flotillin 1 and 2, and annexin II) as well as cytosolic proteins such as ALIX, GTPases (Rab5/Rab7), tumor-susceptibility protein (TSG101) [44], and syntenin (Figure 2A). In addition, exosomes may carry metabolic enzymes, heat shock proteins, and MHC molecules (MHC class I and class II). Due to their enrichment in exosomal cargo, TSG101, syntenin, ALIX, and various tetraspanins have been used as potential markers of exosomes [45, 46]. A recent study by Kugeratski et al. reported results of proteomic analysis of exosomes isolated from different cellular origins and highlighted a cohort of universally enriched 22 proteins, where syntenin1 was the most abundant protein and therefore identified as a potential universal exosome marker [47].

Importantly, Lötvall et al. measured in 2007 the presence of functional mRNAs and microRNAs (miRNAs) in exosomes and showed that exosomal mRNA can be translated into proteins in target cells [10]. Further studies reported the presence of DNA, small interfering RNAs (siRNAs), transferring RNA, small cytoplasmic RNA and mitochondrial DNA, and RNA [10, 48, 49]. Lipidomic analysis of exosomes revealed an abundance of sterols (cholesterol and cholesteryl esters), sphingolipids (sphingomyelin and ceramide), glycosphingolipids, and phospholipids (phosphatidylserine, phosphatidylcholine) within exosomes compared to their releasing cells [10].

After release, exosomes can transmit information to the same cell (autocrine function) or target neighboring or distant cells, herewith exerting their intercellular communication function. There are two generally accepted ways of EV-based cell-cell communication. They can bind to the surface of the recipient cell and initiate intracellular signaling pathways, or they can be internalized by target cells and release their molecular cargo. Cells can internalize exosomes by clathrin-dependent endocytosis, pinocytosis, phagocytosis, and caveolin-mediated endocytosis. The discovery of such compositional variety in proteins and nucleic acids has led to an increased interest in exosomes as mediators of intercellular communication and pathogenesis over a diverse range of cell types.

### 2.2 Microvesicles

The outward budding of the plasma membrane causes the formation of particles 50–2000 nm in diameter known as microvesicles (also known as ectosomes, oncosomes, or shedding vesicles) (Figure 2B) [50]. While significant effort has been dedicated to the understanding of the biogenesis of exosomes, less is currently known about microvesicles.

It has been shown that the release of microvesicles depends on the lipid content of the plasma membrane of releasing cell, as well as on the intracellular calcium concentration [51]. Elevated levels of Ca^+2^ stimulate rearrangement of the phosphatidylserine from the inner leaflet of the plasma membrane to the cell surface result in plasma membrane curvature and cytoskeletal changes. This further supports membrane pinching and the formation of microvesicles. This described process of microvesicles formation is regulated by several enzyme groups that regulate the membrane asymmetry such as aminophospholipid translocases including flippases and floppases, scramblases, and calpain [52]. Some studies showed the importance of lipid rafts, cholesterol-rich domains of the plasma membrane, in microvesicles formation [53]. Another described way of microvesicles formation involves interaction between TSG101 and arrestin domain-containing protein 1 (ARRDC1) [54]. The molecular cargo of microvesicles includes enzymes, signaling molecules, miRNAs, mRNAs, growth factors, and cytokines. The proposed surface protein markers are annexin A1 and A2 which are found to be abundant in the lower flotation density region of EVs that correspond to microvesicles [49].

Following the release from the cell of origin, microvesicles interact with target cells *via* membrane receptors and either fuse with the recipient cell or trigger signaling pathways in the cell in a contact dependent manner [3]. Therefore, microvesicles function as mediators of intercellular communication by transferring bioactive molecules such as nucleic acids, proteins, and lipids between cells. They have been suggested to be involved in the promotion of angiogenesis, transfer of oncogenic receptor protein, and metastasis [55]. Additionally, microvesicles are shown to be involved in the progression of cardiovascular [56] and neurological disorders [57, 58]. This further suggests their possible clinical application as biological markers of the aforementioned diseases.

### 2.3 Apoptotic bodies

Apoptotic bodies (ABs) are a class of highly diverse EVs that can range in size from 50 nm to 5000 nm and have variable morphology. They are formed during the apoptosis of cells and contain cell degradation products such as organelle fragments, DNA, histones, and cytoplasmic components. Apoptotic bodies clearance is performed by professional phagocytes or by neighboring cells [59].

Little is known about their functions, yet it is clear that the formation of ABs promotes the efficient removal of cell debris and may regulate cell-cell communication. Their role in intercellular communication is yet to be explored but it is suggested that they contribute to the cell–cell communication by delivering their molecular cargo. Another important function of ABs is the clearance of apoptotic cell residues without triggering inflammatory reactions. They have remodeled membrane structure where the phosphatidylserine lipid is exposed onto the outer leaflet, and this serves as an “eat me” signal for phagocytes. This leads to a rapid clearance of ABs and prevents secondary necrosis. Altered clearance of ABs has been found to contribute to autoimmune disorders [60, 61]. While ABs have important biological functions, we will not discuss their characterization in the context of this review article.

### 2.4 Exomeres

Recent studies reported the discovery of a new member of EVs termed exomeres [46]. Exomeres are nanosized EVs that are typically less than 50 nm in size and unlike other types of EVs do not have lipid membrane [62]. The biogenesis pathway of exomeres formation and molecular mechanisms of their secretion remains unclear. Proteomic analysis of exomeres showed the presence of proteins related to endoplasmic reticulum, extracellular matrix, mitochondria, cytoskeleton, and higher levels of metabolic proteins. On the contrary, proteins associated with the plasma membrane and exosomes biogenesis pathway were found to be depleted [46, 62]. Exomeres lipid content includes ceramide, diglyceride and triglyceride, and phospholipids such as phosphatidylcholine, phosphatidylethanolamine, and phosphatidylserine [46, 62]. Exomeres are found to carry a nucleic acid cargo of DNA, RNA, and miRNAs [46, 62]. The role of exomeres in cell-cell communication is yet to be determined. Initial studies suggest that they may have a role in the regulation of metabolic pathways of the recipient cells [46, 62]. Due to the lack of studies, in this review, we will not discuss characterization of exomeres *via* label-free optical methods.

### 2.5 MISEV2018 guidelines

The ISEV notes that the size and amount of EVs make them difficult to obtain as pure preparations or to characterize. Specifically, defining functions of specific types of EVs or EVs in general requires comprehensive reporting and testing of potentially confounding properties or variables that arise in impure and heterogeneous samples of EVs. For example, claiming pathophysiological roles of EVs or proposing clinically relevant biomarkers requires rigorous controls of experiments and characterization that are not universal for all EVs types or populations. The ISEV has noted that some articles propose conclusions that are not fully supported by the reported information or experimental design. To better inform researchers and reviewers on designing experiments and processing of EVs, as well as to ensure that reports are sufficiently thorough and repeatable, the ISEV has published the MISEV2018 guidelines, based on the consensus of ISEV scientists. The MISEV2018 guidelines include information related to nomenclature, collection and pre-processing, EVs separation and concentration, EVs characterization, functional studies, and reporting, and are presented in three subcategories: quantification, bulk characterization, and single EV characterization [14].

In terms of EVs characterization, it is important to use multiple, complementary techniques to attest that investigated biomarkers are coming from EVs and not from other contaminants of the isolation process. Quantification of EVs requires some minimal information such as the total initial volume of biofluid, or the number of cells for conditioned medium, the particles number, the total protein, lipid, and RNA amount, or quantification of specific molecules such as tetraspanins CD9, CD63, and/or CD81 or disease-specific proteins. As for bulk characterization, it is recommended to determine expression of three classes of protein markers to prove the presence EVs and their purity: (i) transmembrane or GPI-anchored protein localized in cells at the plasma membrane or endosomes whose presence demonstrates the lipid-bilayer structure of EVs, (ii) the presence of cytosolic or periplasmic proteins able to bind to membranes, and (iii) the presence of protein constituents of non-EV structures often co-isolated with EVs which indicate the purity degree of EVs. Additionally, for small EVs subtypes, the presence of proteins localized in/on intracellular compartments of origin cells has to be evaluated. Single EVs characterization on the other hand requires techniques that allow the visualization of single EVs, such as transmission electron microscopy (TEM) by contrast with uranyl acetate, for example, cryo-EM, scanning electron microscopy (SEM), scanning probe microscopy including AFM or super-resolution microscopy. Another approach for single particles analysis is based on measuring biophysical parameters of single EVs and quantification of large numbers of particles. For example, nanoparticle tracking analysis, high-resolution flow cytometry, multi-angle light scattering combined with asymmetric flow field-flow fractionation (AF4-MALS), fluorescence correlation spectroscopy (FCS), or Raman tweezers microscopy are capable to assess the chemical composition of EVs.

## 3 Particle counting, sizing, and morphology characterization

The morphology of EVs is determined by a variety of parameters, such as specific biogenesis pathways and disease states. To elucidate EVs’ functions and roles in physiological and pathological processes such as cancer and neurodegenerative diseases, it is important to accurately measure and quantify morphological features.

Various techniques have been adapted to enable EVs counting, sizing, and morphological characterization. A recent survey in 2016 showed that both single particle tracking methods (72% out of 196 samples) and flow cytometry (41%) are prevalent among studies, while 9% of the research employed AFM [13]. The majority of EV-related studies used several complementary techniques, with only 9% reporting the use of only one characterization method [13]. It is very difficult to acquire both reliable and multiplexed results when analyzing different types of EVs with a single analysis method, as no current technique can fulfill the complete spectrum of EVs properties of all sizes in polydisperse samples. Despite the attempts for EVs characterization, overlapping biophysical characteristics and variable compositions are still hindering the monitoring of EVs dynamic processes, especially at single-particle level [63]. Thus, novel detection methods are required to study the physical characteristics of EVs. This section describes the general working principle of five commonly used characterization techniques: NTA, DLS, AFM, flow cytometry, and SP-IRIS (Table 1). Several examples of their implementations in studying label-free EVs are provided to further demonstrate their applications. Advantages and limitations are discussed as well.

### 3.1 Nanoparticle tracking analysis

Nanoparticle tracking analysis (NTA) is a technique that can characterize the size distribution and concentration of various nanoparticles in solution or suspension, such as protein aggregates, liposomes, and other nanosized colloidal particles, including EVs [69, 70] Although it is often compared to DLS, which was discovered first, NTA has become the most popular method in single particle tracking of EVs studies [13] due to its ability to simultaneously characterize large particles numbers. The inherent measurement condition keeps EVs in their close-to-native environment, such that artefacts of particle shrinking can be avoided and the sample can be recovered after the experiment [70, 71].

NTA is based on light scattering and Brownian motion of particles in liquid suspensions. It takes advantage of the relationship between particle velocity and hydrodynamic radius to determine a particle’s size through the Stokes–Einstein Eq. (1) [69]:

(1) $$\bar{{\left(x,y\right)}^{2}}=\frac{2k_{B}T}{3r_{h}\pi \eta }$$

where $k_{B}$ is the Boltzmann constant, η is the medium viscosity, $\bar{{\left(x,y\right)}^{2}}$ is the mean-squared speed of a particle, $r_{h}$ is the particle’s hydrodynamic radius, and T is the absolute temperature. This relationship indicates that smaller particles move faster than larger ones.

**Figure 3:**
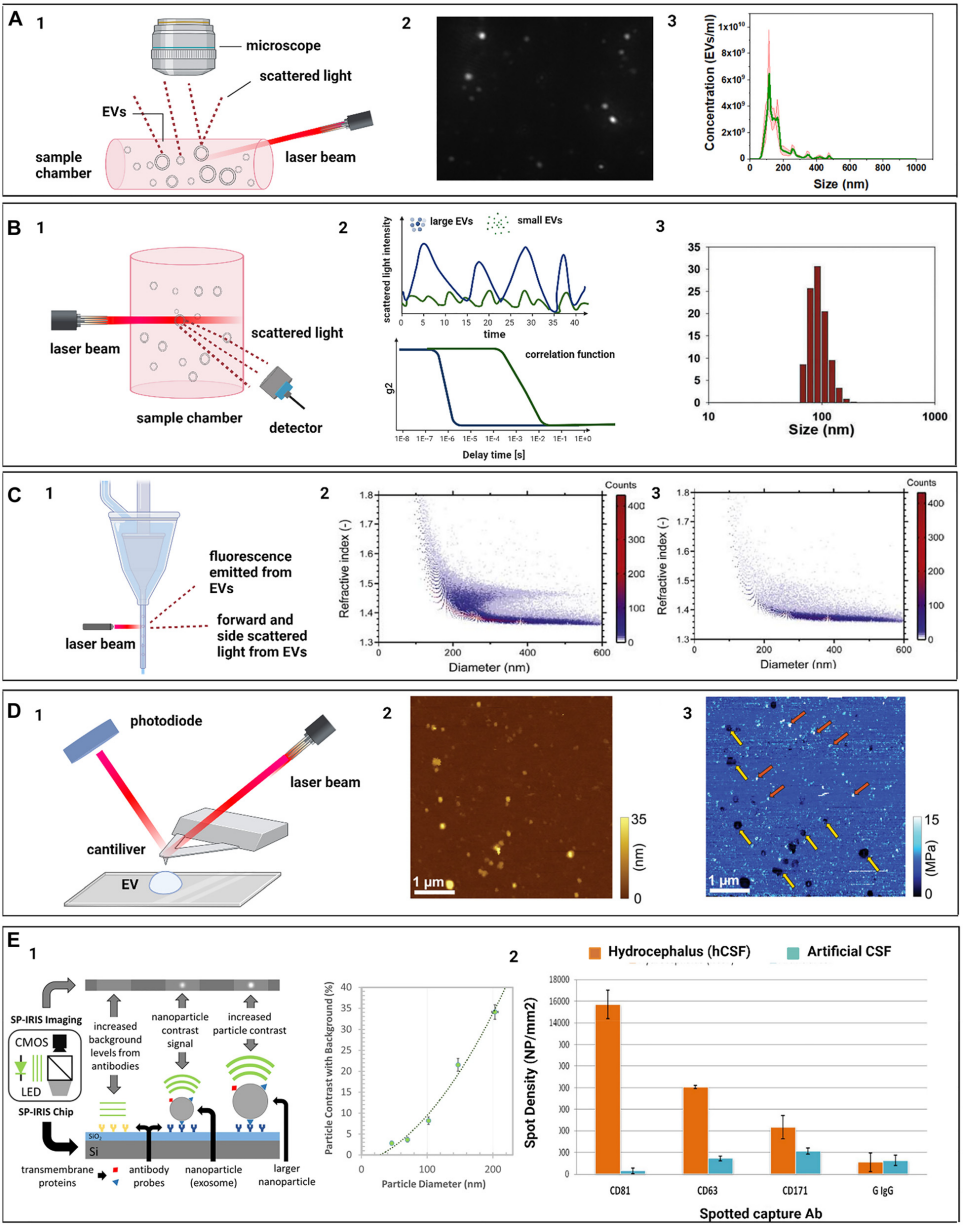
Label-free optical methods for EVs physical characterization. (A) Nanoparticle tracking analysis; (A1) basic setup of the NTA measurement system; (A2) typical micrograph of EVs in the imaging chamber; (A3) graphical representation of NTA histogram of neuronal EVs (adapted with permission from [64]). (B) Dynamic light scattering; (B1) schematic of the optical configuration; (B2) representation of DLS results of scattering of small and large EVs, and their correlation function; (B3) DLS histogram of particle size distribution (adapted with permission from [65]). (C) Flow cytometry; (C1) depiction of working principle; (C2) scatter plot of diameter versus refractive index of EVs and lipoproteins; (C3) scatter plot of all CD61 positive EVs from the same sample (adapted with permission from [66]). (D) Atomic force microscopy; (D1) AFM general components and operating principle; (D2) analysis of mesenchymal stem cells (MSCs) EVs. Topography image shows particles of various sizes. (D3) elasticity map of the same sample area depicted in (D2). Elastic modulus measurements revealed the presence of two types of particles EVs (yellow arrows) and nonvesicular particles (red arrows) (adapted with permission from [67]). (E) SP-IRIS; (E1) schematic illustration of SP-IRIS detection mechanism. The SP-IRIS signal is based on interference of light scattered from the Si–SiO_2_ sensor surface and captured EVs. Graph shows size-dependent correlation of the contrast of particles; (E2) The expression of exosome surface proteins CD81, CD63, and neural adhesion protein CD171 quantified by SP-IRIS against the G IgG control (adapted with permission from [68]).

During NTA, particles suspended in liquid are injected into the sample chamber. As seen in Figure 3A, a narrow laser beam is directed into the chamber in a dark field or total internal reflection configuration such that the background from the incident light is minimized. Particles within the laser beam’s path scatter its light. The chamber is configured so that videos can be captured with an optical microscope linked to a 2D array camera. The video is generally 30–90 s in length with around 30 fps. The software identifies and then tracks individual particles frame-by-frame to determine their velocity. Using the Stokes–Einstein Eq. (1), the size of each particle can be determined and gathered to produce the overall size distribution of particles in the sample. Knowing the volume of the sample, the concentration of particles can be deduced. This sizing range happens to correspond to the known sizes for the majority of EVs.

The lower size limit of NTA depends on the signal-to-noise ratio of the captured images [71]. Both the camera sensitivity and the amount of light scattered by the particles are thus potential limiting factors. At the lower size limit, which is much smaller than the wavelength of light, the particles exhibit Rayleigh scattering, which can be described by:

(2) $$\sigma_{s}=\frac{2\pi^{5}}{3}\frac{d^{6}}{\lambda^{4}}{\left(\frac{n^{2}-1}{n^{2}+2}\right)}^{2}$$

where $\sigma_{s}$ is the Rayleigh scattering cross-section, d is the particle diameter, λ is the wavelength of light, and n is the ratio of the particle refractive index to the solvent refractive index [71]. For particles with higher refractive index values, NTA’s size limit can reach as low as 10 nm. EVs, however, have low refractive index values of approximately 1.37–1.59 [72, 73], limiting the minimum size that can be determined *via* NTA to around 30–50 nm [71]. As particle size increases to near or above the size of light wavelengths, Mie scattering is exhibited. Mie scattering calculations are more complex than Rayleigh scattering, but the key aspect is that the scattering cross-section drops rapidly as particles decrease in size. Mie scattering produces much more intense light scattering compared to Rayleigh scattering, due to the increasing size of the particle. It also distorts the scattering more towards the forward direction, compared to Rayleigh, whose scatter is relatively homogeneous around the particle. The upper size limitation arises when particles are too large and their corresponding Brownian motion speeds are too slow to be accurately measured, which occurs around 1 μm [71].

Although the size range of NTA for EVs is 30–1000 nm, size heterogeneity in samples can further complicate the analysis of size distribution. First, an appropriate concentration of EVs must be present in the sample, otherwise, the particle density is too high and small EVs will not be discerned in a polydisperse sample. Prior to NTA measurements, samples must be diluted until particles can be observed individually, corresponding to approximately 10^7^ to 10^9^ particles/ml [69, 70]. However, as discussed before with Mie and Rayleigh scattering, larger particles scatter light more intensely than smaller particles, which can have an unintended masking effect over smaller particles and cause an underestimation of small particle concentration [69]. In order to properly characterize size distribution in highly polydisperse EVs samples for their entire size range, the NTA analysis of a sample needs to be performed on multiple dilutions where the camera settings are adapted for the different light scattering behaviors of small and large EVs [70]. Moreover, in certain NTA setups, the suspension can be flown with a fixed flow rate through the chamber to increase the precision and repeatability of the results compared to a static suspension, since a greater number of particles are being analyzed. In this case, the flow settings must be carefully selected as it may impact the accuracy of the measurements [74].

Finally, there are several limitations of the NTA method that are important to consider for specific applications. First, one of the significant disadvantages of NTA is its lack of specificity. NTA cannot distinguish between contaminants, such as protein aggregates, or differentiate between EVs. Therefore, adequate isolation methodologies need to be used. Additionally, the precision and accuracy differences of various commercially available NTA machines have been compared, and it has been shown that EVs sized less than 60 nm cannot be accurately detected by several machines [75]. NTA also requires large sample volumes, which can limit its use for studies where a limited amount of sample is available, such as noninvasive clinical applications. It is worth noting that NTA is compatible with fluorescence detection if appropriate markers exist. Although this may increase the resolution and adds more specificity, details are out of the scope of this review. While being broadly applied in EVs sizing and counting, part of the focus for NTA studies has also been put into the optimization of imaging parameters and the standardization between different systems. Comparative studies have been carried out between devices from either the same or different manufacturers, and the need for a standardized protocol still has to be fulfilled.

### 3.2 Dynamic light scattering

Dynamic light scattering (DLS) is a technique that can characterize the size distribution of nanoparticles in solution or suspension [76]. The first device that used these principles to establish the diffusion coefficient of particles in suspension was developed in 1964 [77]. Given its simplicity in sample preparation and operation, accompanied by the need for small sample volumes and fast experiment results of only a few minutes, DLS has become a convenient method in the analysis of EVs size [78].

Dynamic light scattering is similar to the NTA method as they both involve light scattering from particles in suspension. A laser is sent through a dark sample chamber with particles in suspension, as seen in Figure 3B. Particles within the path of the beam scatter the incident laser light. For DLS, this scattered light is often collected by a photon-counting detector [79]. Due to the Brownian motion, particles move in and out of the path of the laser beam and the number of scattered photons recorded by the detector fluctuates accordingly. Essentially, the intensity of light measured by the detector fluctuates as the particles undergo Brownian motion.

The diffusion behavior of nanoparticles is described by the translational diffusion coefficient $D_{\tau }$ , which will allow for the measurement of the hydrodynamic radius *R*_h_:

(3) $$D_{\tau }=\frac{k_{B}T}{6\pi \eta R_{h}}$$

where $k_{B}$ is the Boltzmann constant, T is an absolute temperature ,η is the viscosity of medium, and $R_{h}$ is the hydrodynamic radius. This coefficient can be measured from the decay constant of the normalized correlation functions that describe how the scattering intensity patterns for one particle as well as for particles relative to each other are correlated [77]. For polydisperse particles, these correlation functions provide the link between the intensity decay constant Г and the diffusion behavior $D_{\tau }$ of particles:

(4) $$Г=-D_{\tau }{\left(\frac{4\pi n}{\lambda }\sin\left(\frac{\theta }{2}\right)\right)}^{2}$$

and where λ is the wavelength of incident light, n is the solvent refractive index, and θ is the detector angle. To fit the correlation function to polydisperse samples, a constrained regularization method for inverting data (CONTIN) method is commonly used.

DLS is capable of detecting particles in the range between 1 nm and 6000 nm range [80] and determining their size distributions. This wide detection range makes DLS an adequate fit for sizing EVs. In fact, various recent studies have adapted DLS for measuring the size distributions of the vesicles of interest [81–84].

However, this size range can be strongly affected by statistical errors from the experiments [85]. Particles in DLS analysis follow the same Rayleigh and Mie scattering behaviors as for NTA. Particles much smaller than the wavelength of light exhibit Rayleigh scattering, but as their size increases to the same regime as the wavelength of light, they can be described by Mie scattering [76]. With Rayleigh scattering, as shown in Eq. (2), the intensity distribution depends on the diameter of the particle to the sixth power, making DLS more sensitive to larger particles, as they have more intense light scattering behavior as their diameter increases. DLS is thus particularly vulnerable to potential contaminants and polydisperse solutions [78, 82, 86]. For example, when both DLS and NTA were used to characterize HTBE and Calu-3 vesicles from two airway cell culture systems, the latter technique indicated slightly smaller values for both groups. This further proves that the DLS results can be biased towards larger particles [87]. The same tendency for DLS was observed when assessing the size distribution of polystyrene beads with a range of known sizes, with the larger end of particle sizes being slightly larger than the NTA results [88]. Moreover, natural aqueous samples with a high polydispersity index can lead to misleading size distribution data in DLS [89]. This also implies that DLS has rather a low peak resolution compared to other techniques discussed in this section, given that it failed to produce bimodal distributions for a mixture of 20 and 100 nm particles [69, 90]. Lastly, being a low-resolution technique, DLS cannot further distinguish between similar-sized EVs, as it was unable to differentiate microvesicles apart from lipoprotein particles or small platelets when studying the selective release of circRNAs [91, 92].

Since DLS is inherently incompatible for more quantitative measurements, such limitation can be partially complemented with Bradford assay to recover sample concentration [82, 84], or size exclusion chromatography to perform sample fractioning first [93]. Other options of using DLS to determine the presence of specific surfaces markers may include its combination with the immunoprecipitation approach [94].

Being a non-invasive and label-free technique with relatively high accuracy, DLS is often used along with flow cytometry as a complement for vesicle sizing or as validation for particles sizes acquired with NTA [69]. For instance, such complementary approaches were used to study human adipose-derived stem cell EVs on delaying cartilage degeneration, as well as examining the promotion of cancer lung metastasis upon treatment with indoor dust EVs [65, 95].

### 3.3 Atomic force microscopy

Atomic force microscopy (AFM) is a technique that can characterize the topography, size, and a number of physical and mechanical properties of a material’s surface [96]. AFM was developed in 1985 and in the past years has been widely applied in the biology research field.

AFM is a type of scanning probe microscopy technique with the probe that consists of a sharp tip attached to the end of a flexible cantilever [97, 98]. As the tip glides over the sample surface with or without contact, the distance between deposited samples and the tip is monitored based on the cantilever deflection or other feedback parameters (Figure 3D), depending on the selected mode. The recorded vertical positions can be later used to reconstruct the 3D topography of a sample or as a probe for other mechanical properties [99, 100]. AFM has great advantages over other morphology characterization techniques for being the only laser-based technique capable of identifying EV morphology and topography at nanometer-scale resolution [101]. Furthermore, AFM can be conducted under physiological conditions and is non-destructive (depending on the mode of operation). Despite the unsurpassed size resolution and level of morphological and physical detail, AFM’s primary drawback is its low throughput. AFM requires extensive labor dedicated to only one particle at a time. AFM is therefore a useful technique for validation of other characterization methods but is not used in high-throughput applications.

AFM can be operated in various modes, depending on the specific scientific question that needs to be answered. The specific modes and their use for characterizing EVs are outlined below.

The *contact mode* through keeping a constant tip height or deflection can easily measure the mechanical properties of the surface with a force-distance curve, but it may bring irreversible deformations and damage to samples with the probing forces being hard to control [102]. Previous studies also found that mechanical stimulation of AFM could increase the release of exosomes as an important stress response [104]. There are several other AFM modes that are more suitable for soft biological samples and have been applied in various EV-related studies.

First is the *tapping mode* in which the cantilever vibrates near its resonance frequency and only encounters the surface intermittently. The tip-surface interactions are significantly reduced in the lateral direction and less perturbation is brought to the vesicles. It is now one of the most commonly seen modes in EVs characterization and has been used to compare the size distribution of salivary and conditioned cell media EVs under aqueous conditions [104]. The same application in finding EVs morphological properties of shape visualization and sizing can be seen elsewhere [101, 105, 106]. Although EVs counting is more often carried out with light scattering techniques discussed in previous sections, AFM with an intrinsically high spatial resolution (>0.5–1 nm) is capable of collecting particle numbers as well. The result is presented as EVs densities in the unit of number of particles per unit area [84, 88, 107].

A more advanced mode, *peak force tapping (PFT) mode*, also known as peak force QNM in quantitative mode, directly controls the deformation depth with the applied forces minimized to only a few piconewtons. With individual force curves being acquired with each tap, not only morphology, but also quantitative sample properties such as adhesion, deformation, and modulus can be mapped simultaneously. Sample stiffness, elasticity, and even energy dissipation can be calculated from the acquired data [107, 108]. However, this mode is still infrequently used for EVs characterization and only a few studies have employed it for quantifying EVs rigidity or adhesive properties [94, 107, 109]. Another variation of this technique named *phase modulation AFM (PM-AFM)*, allows the mapping of compositional differences across the sample surface by recording the phase shift between the excitation force and the tip response. The phase shift values at a fixed feedback amplitude are converted into energy dissipation, which in turn can be used to characterize the adhesion and viscoelasticity of the sample surfaces [108]. Studies that utilize this mode for EVs studies are not commonly seen, yet a recent study published in 2020 applied PM-AFM to quantify physical heterogeneity among several EVs populations [110].

*Nanoindentation* is a technique related to AFM and its uniqueness lies in the ability of probing mechanical properties (stiffness) of submicrometer-sized vesicles (>20 nm) quantitatively. As the probing tip directly presses onto the surface of particles in the vertical direction, the indented distance is recorded when a pre-set peak force value is reached. The sample rigidity can be later on calculated from the function that relates deformation to the applied force [111–115]. Raman techniques: fundamentals and frontiers Consequently, it is often used as a complement to AFM as the latter focuses more on the morphological properties [116]. By following well-established protocols, nanoindentation has been an effective method both in investigating the relationship between liquid composition with vesicle mechanics and their vesiculation pathways [102, 114, 117]. A recent study reported the use of AFM to distinguish EVs from non-EVs particles within the same EVs isolate based on their mechanical properties, specifically Young’s modulus (elasticity) [67]. The one subpopulation of the isolated MSC EVs showed a low Young’s modulus value (4.5 ± 2.0 MPa) that corresponds to higher flexibility and has been reported previously for EVs (0.2–2.7 MPa). On the other hand, the second subpopulation showed a higher Young’s modulus value (18.8 ± 10.8 MPa,) which relates to less elastic particles. It is important to note that morphological characterization of the isolated EVs by AFM was not able to distinguish EVs from non-EVs particles due to their similar size and height. Aside from selecting the appropriate imaging mode, acquisition mode and surface immobilization are all important factors to be considered when studying EVs. AFM is compatible with both air [123] and liquid imaging environment [107, 118] in EVs studies, with the latter being more recommended. Although the air condition is more stable with immobilized EVs, it may cause an underestimation of sample sizes due to inevitable drying when the samples are exposed to the air, leading to mechanical artefacts. Indeed, the typical “cup shape” morphology resulted from EVs dehydration can often be observed when imaged in the air [119]. Conversely, the liquid mode resembles more of the EVs’ physiological conditions, and the native spherical shape can be preserved to allow more accurate size measurement. AFM imaging of dry samples is useful when it is needed to rapidly check the presence of the EVs. However, AFM characterization of EVs in the liquid will yield in more profound size distribution and mechanical properties assessment [120].

As a fairly new member in the field of EVs characterization, this multiparameter technique does not come without limitations. It is highly labor-intensive and only analyzes one particle at a time, making it a low-throughput method. Moreover, aside from the mechanical stress caused by tip-surface interactions, the tip convolution effect often leads to lateral expansion of particle sizes, especially when the probed features are smaller than the cantilever tip size. Additionally, EVs binding to the flat immobilization surface also experience deformation and alter their geometry accordingly [118, 114, 121]. In a 2013 study that explored cell-to-cell communication *via* exosome-like particles, the observed widened vesicle dimensions were likely to be a combined effect of the factors stated above [122]. Nevertheless, a deconvolution algorithm has successfully reduced the widening error, and the tip geometry can possibly be addressed by depositing nanobeads with known dimensions during measurement [121, 123]. Moreover, innovative correlation techniques such as AFM combined with infrared spectroscopy (AFM-IR) or Raman spectroscopy may provide new insights into resolving heterogeneity among EVs populations [96, 124] .

Although primarily being a label-free method, both antibody surface immobilization or gold nanoparticle (AuNP) labelling methods enables AFM to locate and measure certain groups of EVs more precisely [106, 107, 121]. However, detailed discussions regarding labelling approaches are outside the scope of this review.

### 3.4 Flow cytometry

Flow cytometry (FC) is a well-established, high-throughput technique of particle analysis. It can capture a variety of chemical and morphological information. Specifically, for morphological characterization, flow cytometry can measure particle size and count using light-scattering principles [125]. Conventionally, flow cytometry requires larger sample volumes, but it has recently been shown to work for microvolume samples [126].

A typical flow cytometry instrument relies on three systems: the fluidics system, the optics system, and the electronics system. As seen in Figure 3C, the fluidics system collects the sample liquid and controls the flow of the stream so that the individual cells or particles are focused into a single-file path in the center of the stream. At the interrogation point, a laser light normal to the stream illuminates the individual particles, who scatter the light. The scattered light is filtered and collected by the optical system with sensors perpendicular to the stream path in the forward and side directions. Finally, the electronics system receives the signals collected by the optical system and processes them to output the collected data [125, 127, 128].

The fluidics system is responsible for organizing the flow of the particles such that they can individually be illuminated by a laser and their scattered light read by the sensors. To do this, the fluidics system uses an additional sheath fluid to hydrodynamically focus the sample fluid. The sheath fluid is a simple saline solution that is pumped with constant pressure from a chamber through a narrow nozzle. The sample fluid is pumped through an inlet tube through the center of this chamber and meets the faster-moving sheath fluid as they both enter the same nozzle, causing the sample fluid to be forced into a smaller, central core stream that passes through the nozzle with the sheath fluid surrounding it. The drag that occurs at the boundaries of the sample stream as it enters the sheath fluid stream directs the particles in the sample fluid towards the middle of the stream, where is a faster and more stable flow. This central core containing the particles from the sample liquid will have laminar flow and the particles will travel at approximately the same speed through the same single axis. Without this focusing effect, the cells or particles in liquid would move in a disorganized and turbulent manner, introducing variability to the data. Furthermore, their proximity or overlap could cause two particles to be read at once by the optical system. The differential pressure between the sample and sheath fluid controls the width of the core stream. The lower the differential pressure, the narrower the core stream, which allows cells or particles to pass through the interrogation point single file on the same axis. Increasing the differential pressure by increasing the sample pressure will allow for a higher flow rate and faster processing times, but will cause widening of the core stream, which could cause multiple particles to line up beside each other and pass through the interrogation point at the same time [125, 126, 128].

To record an event, the signal received by the sensors must pass a specified “trigger” threshold, above background noise. This trigger can be based on three different modes: forward scatter, side scatter, or fluorescence. Often, particles are labeled with fluorophores, and multiple dedicated lasers within the instrument will excite them and the fluorescent light will be collected. The forward scatter light (FSC) (0.5–5.0°) and the side scatter light (SSC) (15–150°) are generally considered to be indications of the particle size and granularity, respectively [127]. However, light scattering depends heavily on a variety of other parameters, including the refractive indices of the sheath, sample media and particles, the laser wavelength, contact and collection angles [127]. It is important to consider that FSC signals are highly variable between different instruments [127].

Furthermore, SSC can also be correlated with size, especially for smaller particles. The Mie theory predicts a strong dependence of light-scatter intensity on the angle of measurement [128]. For EVs that are smaller than the wavelength of light, scattering is more isotropic. Thus, given a lower background in the SSC direction, it has been shown to be more effective at capturing the scattered light.

It is well known that size limitations for conventional flow cytometers render them insufficient for EVs characterization, given that they were first designed for cell analysis. The lower detection limit is usually around 300 nm, meaning the majority of EVs ranging from 30 to 300 nm are undetectable. Nevertheless, instrument setups have been adapted to accommodate small particles and heterogeneous samples. Both high throughput and single particle analysis are attractive properties of flow cytometry in further understanding EVs morphological and functional diversity. As discussed earlier, light scattering for smaller particles is less intense. Detection of such low intensity light scattering events is challenging due to low signal-to-noise ratios [125, 129].

For conventional flow cytometers that were developed for cells, the core stream and laser beam dimensions would allow hundreds of EVs to fit within the interrogation point instead of a single particle [130]. The combined signals of these particles reach the detection limit and trigger the event to be screened as a single particle. This phenomenon has been termed as “swarm detection” and leads to the underestimation of EVs counting [131]. To validate single particle detection, researchers often use serial dilution to observe how the fluorescence or light intensity changes with controlled concentration changes [133, 132].

Adapting flow cytometers for EVs characterization encompasses a variety of hardware modifications. Higher laser power and lower wavelength lasers have been used to produce more intense light scattering [66, 132]. Furthermore, narrowing the core stream and reducing the flow rate also benefit EVs characterization [132]. Modifying the FSC and SSC angles used to collect larger angle scatter is also useful because smaller particles tend to scatter light at larger angles, proportional to their overall light scatter [68, 127, 134]. In a few studies that reported the detection limit of a commercial flow cytometer to be around 200 nm, the SSC detector was set to be a photomultiplier tube. When compared with the photodiode commonly used for FSC detectors, this approach is not only more sensitive, but also detects scattered light over a much broader angle, which allows capture scattering from multiple particles smaller than the wavelength of incident light [131, 135, 136]. Finally, using more sensitive detectors by, for example, incorporating avalanche diodes, can also increase the signal-to-noise ratio of the setup [130].

Impedance-based flow cytometry is different from all the above. It is a non-optical, label-free technique based on the Coulter principle and can be used for the determination of both EV size distribution and concentration. As vesicles flow through the narrow sensing aperture under an electric field, a voltage pulse is generated across the aperture whose amplitude is proportional to the volume of the particle. Although it is only capable of resolving EVs above 300 nm, its unique compatibility with microfluidic devices among flow cytometry enhances the portability and further extends potential applications [137, 138]. So far, it has been applied to characterize EVs bound to extracellular matrix molecules [139], as well as the profiling of miRNA of exosomes from peripheral blood samples [140] (Table 1).

### 3.5 Single particle interferometric reflectance imaging sensor

Single particle interferometric reflectance imaging sensor (SP-IRIS) is an interferometric imaging-based technology for individual particle detection [143, 144]. The signal generated during the measurement is an interference between the scattering signal from the particle of interest and the signal reflected from the layered substrate [143], as shown in Figure 3E1. The typical SP-IRIS system consists of a monochromatic LED, Si-SiO_2_ sensor chip, and CCD camera. The SP-IRIS signal is mainly affected by the polarizability of the particle, amplitude of the reference field, and the phase lag between them. The phase shift of the scattered and reflected light is regulated by the thickness of the SiO_2_ layer of the sensor chip. The surface of the sensor chip is typically immobilized with capture antibodies of the proteins of interest. The technology has been used for quantification and size characterization of viruses in the serum or whole blood [145]. SP-IRIS shows great potential to be applied in the field of EVs characterization due to its ability to detect surface molecules and to measure the size of single EVs [68, 144]. The method had been applied to measure the size of individual EVs derived from cerebral spinal fluid and their surface protein profile [68] (Figure 3E2). Moreover, a recent study employed SP-IRIS to explore tetraspanins expression profile across single EVs from cell culture media and ovarian cancer patients’ serum [142]. The authors demonstrated the uneven distribution of CD9, CD63, and CD81 tetraspanins commonly used as EVs capture markers. These results shine light on the heterogeneity of EVs and may impact the EVs diagnostic application.

Current limitations of the SP-IRIS method are its inability to measure particle concentration [146] as well as the potential detection of several vesicles instead of single particles due to the diffraction limit of optical microscopy (lateral resolution approximately 200-400 nm).

### 3.6 Super resolution microscopy

Super resolution microscopy (SRM) offers a unique opportunity to characterize samples with a size that is below the light diffraction limit, reaching spatial resolution capabilities as low as tens of nanometers. The most popular SRM techniques are based on fluorescence signal detection [147]. However, these methods have several drawbacks including phototoxicity and photobleaching. Label-free SRM, on the other hand, is a more desirable option in the biomedical research field and has a wider range of applications compared to fluorescence-based techniques [148].

Currently, there is no reported research that employed label-free SRM techniques for EVs characterization. Yet, several studies applied label-based SRM in EVs research for EVs tracking [149] and cancer diagnosis immunoassay [150]. Using SRM, specifically direct stochastic optical reconstruction microscopy (with lipid dye), Nizamudeen et al. detected 20-30 nm size EVs [141].

## 4 Characterization of EVs molecular content

The molecular composition of EVs includes nucleic acids, lipids, proteins, and various other biomarkers. EVs released from healthy and diseased cells are known to carry a cargo with different molecular compositions. Therefore, the biochemical content of EVs circulating in body fluids is useful for the evaluation of a patient’s health and early diagnosis of diseases. However, the concentration of EVs released by diseased cells that are present in body fluids is minimal compared to the concentration of EVs originating from normal cells. Therefore, it is crucial to translate and adapt innovative and accurate methods for the analysis of EVs. Traditional methods for EVs’ molecular content characterization are mass spectrometry, Western blot, ELISA, and qPCR. However, these methods require large volumes and high concentrations of EVs, which is a major drawback for many applications. Recently, label-free optical methods are being employed for the analysis of EVs’ molecular composition since they provide several advantages over the traditional methods. Raman spectroscopy, SERS, SPR, and IR spectroscopy are able to identify surface receptors and membrane proteins, in addition to EVs internal molecular cargo (Table 2).

In the following sections, we will review some of the most recent studies reported on the EVs molecular characterization using the aforementioned methods.

### 4.1 Raman spectroscopy

Raman spectroscopy is a label-free spectroscopy technique based on inelastic scattering of laser light that interacts with molecular vibrations. When incident photons interact with molecules, some of the photons are scattered with particular energy shifts, as a function of the structure and composition of the sample [151]. The frequency of scattered photons is recorded and translated into a Raman spectrum that contains the fingerprint of analyzed samples with information on their molecular composition [152]. Since it is a nondestructive and label-free technique, Raman spectroscopy is an ideal tool to investigate EVs. As we mentioned before, EVs are released by all mammalian cells and travel the entire body through the stream of body fluids. They are specific and resemble the cells of origin, carrying a molecular cargo that includes a variety of molecules, proteins, and nucleic acids that may also potentially serve as disease biomarkers relevant for liquid biopsy [8]. With no need for selective antibodies or labels that can interfere with the sample signal, Raman spectroscopy could potentially record even small differences or alterations in the EVs membrane or molecular content. For the analysis of such subtle variations, Raman spectroscopy is often complemented and empowered by multivariate analysis techniques, such as principal component analysis (PCA). These methods are able to analyze and interpret high-dimensional data sets to demonstrate spectral differences between different EVs populations [153].

Since the first Raman spectrum of EVs was reported in 2009 [154], Raman spectroscopy has been extensively employed for the study of EVs. Next, we will review some of the most recent advances in the detection, analysis, and investigation of EVs composition by Raman spectroscopy.

Raman spectroscopy has been employed to characterize EVs in bulk [158–160] as well as single EVs [155–166]. Bulk characterization of EVs *via* Raman spectroscopy showed differences in tissue specific biochemical composition of EVs and the ability to distinguish EVs based on their Raman spectra with high accuracy (>90%) [161, 159]. Moreover, Raman spectroscopy of EVs measured in bulk is a fast and sensitive method to assess EVs purity after isolation [160]. On the other hand, Raman spectroscopy is a powerful tool to characterize the biochemical content of the membrane and cargo of single EVs, and therefore may unravel the heterogeneity of EVs [155, 162, 164, 165]. Such single-particle analysis techniques are essential in the field of biomarkers discovery due to their ability to provide information about the protein, lipid, and nucleic acid content of a single EV [161, 163, 165]. Moreover, the combination of single EV Raman spectroscopy with machine learning algorithms has great potential to be used as a rapid and sensitive diagnostic tool [155, 161, 164, 166].

**Figure 4:**
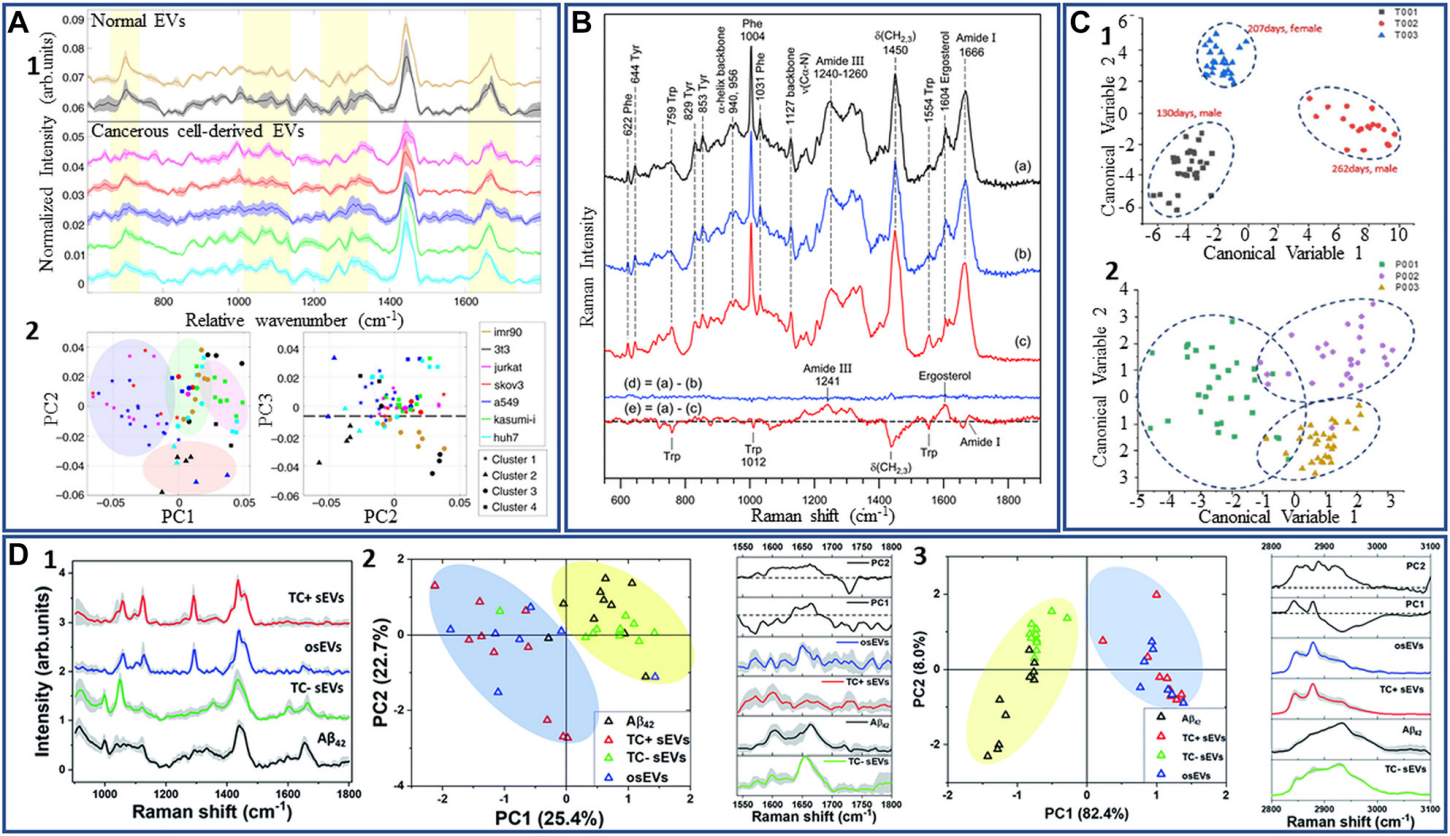
Label-free Raman characterization of EVs. (A) Differences between normal and cancer-cell derived single EVs obtained with Raman tweezer microspectroscopy; (A1) Raman spectra from single EVs derived from healthy and cancerous cell lines; (A2) Scatter plot of PC1 and PC2 obtained for principal component analysis for each cell line. Cell lines are represented with different colors and cluster membership is highlighted by different shapes as shown in the legend (adapted with permission from [156]). (B) Study of Raman tweezers microspectroscopy of EVs. Differences between EVs isolated from cells treated and untreated with hepatotoxin acetaminophen (treated cells - curve a and b, nontreated cells – curve c), as seen in the bottom spectra (reproduced with permission from [157]). (C) Principal component analysis (PCA) – linear discriminant analysis (LDA) of the EVs obtained from peripheral mononuclear blood cells (P001, P002, and P003) and trophoblast cells (T001, T002, and T003); (C1) Plots of trophoblast derived EVs from three different bovines; (C2) Plots of PBMC-derived EVs from three different bovines (adapted with permission from [158]). (D) Investigation of amyloid beta (Aβ_42_) presence in the molecular cargo of EVs isolated from AD cell culture model; (D1) average Raman spectra of TC-sEVs, TC + sEVs, osEVs, and Aβ_42_ pure protein in the fingerprint region; (D2) Analysis of the Raman spectra of the amide I region 1540–1800 cm^−1^. The scatter plot of the first two principal components for each EVs group; (D3) Analysis of the “high-wavenumber region” 2800–3100 cm^−1^ of normalized Raman spectra. The PCA scatter plot of the first two principal components (adapted with permission from [64]).

Using Raman spectroscopy, our lab reported that EVs subpopulations are shared among different cell types, based on their functionality [155]. This report revealed the heterogeneous chemical composition of single EVs isolated from both cancerous and noncancerous cells by employing laser tweezers Raman spectroscopy to trap individual EVs within the laser focus. While there are similarities among the recorded spectra, some major differences can be noticed between EVs derived from healthy and cancerous cell lines (Figure 4A1). These are the higher intensity of the peak centered at 700 cm^−1^, in the case of EVs isolated from noncancerous cell lines, the shape of the 1000–1100 and 1200–1300 cm^−1^ regions, and the 1600–1700 cm^−1^ region, which discriminates 3 cancer lines derived EVs from the others. The PCA spectral analysis of the averaged spectra revealed a significant variation in the chemical content of EVs which clusters into 4 distinct groups corresponding to 4 major EV subpopulations, unspecific to origin cell lines (Figure 4A2). Most of the 7 cell lines analyzed present multiple subpopulations of EVs and all subpopulations contain EVs from multiple cell lines. These differences are mainly due to the membrane content of the EVs. For example, noncancerous cells present mostly 2 subtypes of EVs that are enriched in cholesterol and relatively depleted in phospholipid, compared to EVs isolated from cancerous cell lines.

Also, with Raman tweezers microspectroscopy, the biomolecular content of a small number or even single EVs around 100 nm was characterized in colloidal suspensions. Besides the regular EVs fingerprint biomolecular contributions from proteins, lipids, and nucleic acids, this technique discriminated different subpopulations present in a heterogeneous EV sample isolated from the same cell line. Using Raman tweezers microspectroscopy, Kruglik et al. highlighted the differences in biochemical composition of EVs isolated from rat hepatocytes before and after the treatment with a hepatotoxin [156] (Figure 4B). The results showed some major changes in the biomolecular contents of the EVs, reflected in the Raman spectra by a decrease of the tryptophan content, an enhancement of the amide III band, and a shift of the amide I band to higher frequencies. In addition, the spectral changes included an enhancement of the 1602–1604 cm^−1^ band, assigned to ergosterol, lipids that contributes to the regulation of membrane fluidity, plasma membrane biogenesis and function. Therefore, this technique showed high sensitivity for detection of the hepatotoxicity signatures in EVs and could potentially be applied for the diagnostic of liver damage.

Raman spectroscopy was also successfully employed to characterize and differentiate four types of EVs derived from healthy red blood cells and platelets, and two different prostate cancer cell lines PC3 and LNCaP, respectively [167]. The recorded spectra reveal the distinctive Raman signatures of EVs both in the fingerprint and high-frequency region, with characteristic lipid peaks at 2847 and 2876 cm^−1^, protein contribution at 2932 cm^−1^, CH_2_ deformation in lipids at 1296 cm^−1^, CH_2_ and CH_3_ deformation in proteins and lipids at 1440 cm^−1^, phenylalanine at 1603 cm^−1^, amide II at 1544 cm^−1^ and C=C stretching in lipids 1650 cm^−1^. However, the spectral differences across different EVs subpopulations are subtle, and further PCA was required. PCA analysis was able to clearly separate the Raman spectra into four distinct groups specific to different EVs subtypes. The best separation is obtained in the fingerprint region which provides an obvious discrimination of the EVs groups with a 94.67% and 98% of the data being classified into two categories: healthy cell-derived EVs and prostate cancer-derived EVs.

Recently, Dash et al. used label-free Raman spectroscopy to analyze and compare the EVs isolated from conditioned media of two cancer cell lines (COLO 205 and MCF-7) by three different isolation techniques, namely total exosome isolation reagent, protein organic solvent precipitation, and differential ultracentrifugation [168]. Raman spectra were collected using a 532 nm laser line and then multivariate analysis was performed to study specific variations of the EVs spectra. This allowed the authors to differentiate the isolation techniques and classify them based on the quality of spectra.

Another study published in 2020 showed the potential application of placental EVs as non-invasive liquid markers for pregnancy complications prediction and monitoring. The results of the study determined certain peaks such as 728 cm^−1^ and 1573 cm^−1^ that attribute to collagen and purine/protein can only be found in EVs isolated from PBMC, whereas cholesterol and tryptophan peaks located at 702 cm^−1^ and 1553 cm^−1^ appear only in trophoblast EVs [157] (Figure 4C). Also, the 784 cm^−1^ peak (DNA/RNA) can be found in both EVs types, but it is significantly higher in the spectra of the PBMC derived EVs. The authors used PCA and LDA to analyze data collected from Raman spectroscopy characterization of EVs (Figure 4C1–2). Interestingly, they found no significant difference in Raman spectra of PBMC EVs of different bovines while EVs derived from trophoblast of three placental samples were clustered separately based on gestational age.

Additionally, using Raman spectroscopy, our group highlighted the presence of amyloid beta (Aβ) in the molecular cargo of EVs isolated from the Alzheimer’s disease cell culture model [63]. Specifically, we used EVs derived from MC65 human neuroblastoma cells with the secretion of Aβ regulated by tetracycline promoter. To investigate the presence of Aβ in EVs, we analyzed the EVs isolated from MC65 cells before and after treatment with tetracycline, denoted TC- and TC + EVs, respectively (Figure 4D1). This will ensure that the biochemical difference between the two EVs groups is due to the potential association of Aβ in TC-EVs. PCA analysis and the intense Raman peaks centered at 1650 cm^−1^ and 2930 cm^−1^ together with TC-EVs spectra similarity with the spectra of pure Aβ protein confirm the presence of the Aβ in TC-EVs (Figure 4D2–3). Also, we observed some differences in the lipid structure of EVs. For example, TC + EVs present lipids with longer fatty acid chains while TC-EVs have shorter fatty acid chains, suggesting the association of Aβ protein with plasma membrane which alters the membrane fluidity. The results were validated by testing EVs isolated from 3D midbrain organoids as a healthy brain neurons control.

Collectively, Raman spectroscopy is a useful tool that can complement traditional EVs characterization methods and provide valuable information about the biochemical content of EVs. Yet, inherently weak Raman scattering limits the application of the technique. To overcome this challenge, plasmonic nanomaterials and coherent Raman techniques have been applied to enhance the intensity of the Raman signal.

### 4.2 Surface enhanced Raman spectroscopy

As we showed above, Raman spectroscopy is a highly useful method for EVs characterization. However, during Raman scattering, very few photons are scattered inelastically and as a result, the Raman signal is very weak. Consequently, this requires higher sample concentration, laser power, and a long integration time for the acquisition of quality spectra [170]. However, the Raman signal can be significantly enhanced up to ∼10^14^ times by a technique called surface enhanced Raman spectroscopy (SERS) [170]. SERS technique is based on the phenomenon of surface plasmon resonance. Plasmon resonances from irregular substrates with gaps and junctions created between nanoparticles (NPs), form hot-spots that enhance the signal from Raman active molecules located in these regions. This makes SERS spectroscopy a powerful technique with the capability to detect and analyze even single molecules [171].

**Figure 5:**
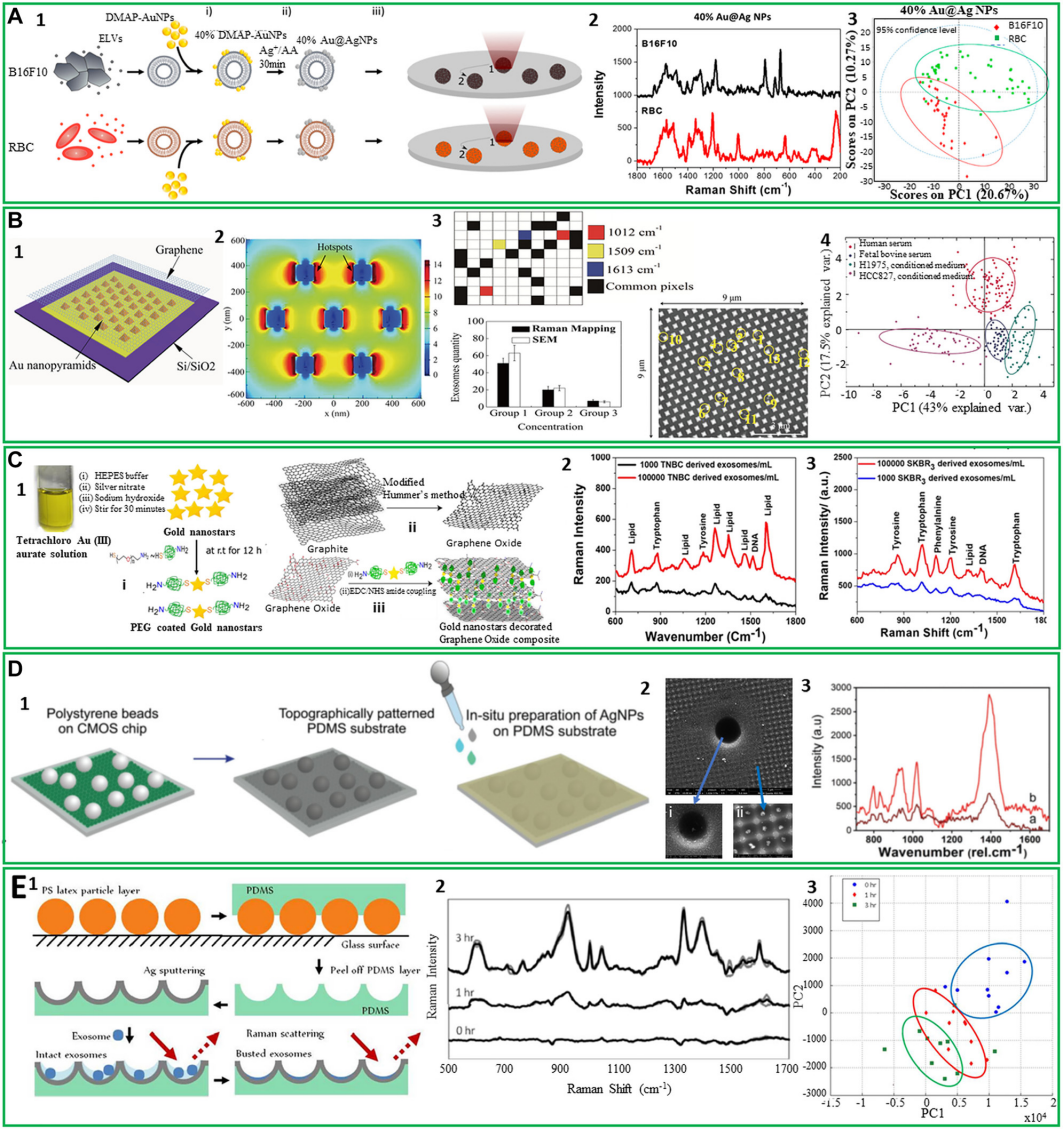
Label-free SERS characterization of EVs. (A) Functionalization and SERS analysis of EVs isolated from two types of cells; (A1) Schematic representation of the procedure for EVs functionalization; (A2) SERS spectra of individual B16F10 melanoma- and RBC-derived EVs; (A3) The 2D PCA for B16F10 and RBC EVs Au@AgNPs (adapted with permission from [175]). (B) Fabrication of a hybrid platform for SERS investigation of EVs; (B1) Schematic diagram of the SERS hybrid platform; (B2) Electromagnetic field distribution simulated by FDTD for 785 nm laser; B3. SERS and SEM mapping of EVs adsorbed on the hybrid substrate; the graph compares the EVs density obtained through Raman mapping and SEM at three different EVs concentrations. SEM micrograph of EVs attached to the graphene-covered surface, where the yellow circles mark the presence of EVs within this region. The colored pixels represent the presence of selected peaks in the Raman spectrum; B4. PCA analysis of EVs from different sources (adapted with permission from [177]). (C) Synthesis and analysis of EVs on a mixed metal–graphene substrate; (C1) Schematic representation of the synthesis of PEG-coated gold nanostars; (C2) SERS spectra of EVs isolated from TNBC cells (10^5^ and 10^3^ cell/ml) on the fabricated surface; (C3) SERS spectra of EVs obtained from SKBR3 cells (10^5^ and 10^3^ cell/ml) on the developed surface (adapted with permission from [180]). (D) Fabrication of a superhydrophobic substrate for EVs concentration and analysis; (D1) Schematic illustration of the substrate fabrication containing micro and nanobowls; (D2) SEM image of the AgNPs grown on the patterned PDMS substrate; insets show the AgNPs grown inside (i) microbowls and (ii) nanobowls; (D3) SERS spectra of EVs acquired with a633 nm laser excitation from inside of (a) nanobowls and (b) microbowls (adapted with permission from [179]). (E) Preparation of an SERS substrate and its use in analysis of intact and ruptured EVs; (E1) Schematic representation of the substrate preparation; (E2) SERS spectra of EVs isolated from SKOV3 cell line recorded at different time intervals; (E3) PCA of recorded SERS spectra (adapted with permission from [180]).

In the field of EVs characterization, SERS is particularly important due to its label-free and nondestructive nature and ability to obtain information about the vibrational modes of molecules with high sensitivity [172]. Additionally, considering the low abundant populations of disease-related EVs compared with EVs from healthy cells in body fluids, SERS overcomes the aforementioned Raman limitations [173]. To date, different types of SERS substrates have been developed for the study and characterization of EVs. Stremersch et al. used an innovative method to characterize EVs *via* SERS. In this study, individual EVs were enveloped in gold shells [174]. In this way, due to the reduced size of the AuNPs, multiple hot spots were created, enabling the recording of an intense SERS signal. This signal is coming from the DMAP molecules that were functionalized on the gold surface, and from the biomolecular EVs components with peaks located at 1123 cm^−1^, (lipids and proteins), 1172 cm^−1^ (proteins), 1307 cm^−1^ (proteins and lipids), 1366–1370 cm^−1^ (phospholipids and carbohydrates), 1445 cm^−1^ (lipids and proteins), and 1572–1576 cm^−1^ (nucleic acids). The authors proved the diagnostic potential of this method by discriminating EVs isolated from B16F10 melanoma cells from the healthy red blood cell (RBC)-derived EVs. Later, this approach was improved by growing a silver layer directly on the surface of EVs coated AuNPs to form core–shell Au@AgNPs directly on the surface of EVs [185] (Figure 5A1). Thus, the interfering signal from DMAP stabilizing molecules is removed and an additional near-field enhancement is obtained from the core–shell structure (Figure 5A2). The spectra recorded using this system allowed a better separation of the cancerous and healthy EVs data points in the PCA analysis and quantification of the discriminative capability of the system with the partial least-squares discriminant analysis (PLS-DA) (Figure 5A3). The performance of this approach was proved by a higher than 90% specificity and sensitivity determined for both EVs types.

Yin and co-authors proposed a new isolation method for cancer cell-derived EVs and a new label-free SERS substrate [176]. Specifically, they used a PEG-based method to isolate the EVs by simply incubating a PEG solution with conditioned media for 24 h at 4 °C and centrifuging the mixture for 15 min at 3000 g. Three types of male cancer cell lines COLO-205 (colorectal cancer), THP-1 (leukemia), and DU-145 (prostate cancer), and healthy male blood samples were selected for EVs isolation. The obtained EVs were placed on an amino molybdenum oxide (AMO) nanoflakes substrate to be detected by SERS under 532 nm laser irradiation and analyzed by PCA supported by vector machine (SVM), a machine learning method able to accurately recognize complex vibrational signals of EVs. This method provides close to 100% accuracy in differentiating the healthy and cancer EVs.

Recently, a new plasmonic hybrid platform based on periodically arranged Au nanopyramids covered by a single layer of graphene was proposed [177] (Figure 5B1). In this setup, the metallic nanopyramids generate hotspots with a high electromagnetic field on each side (Figure 5B2) for enhancement of the EVs Raman signal, while the graphene layer provides a biocompatible and chemically stable surface for EVs detection. To test this label-free SERS substrate, EVs from four different sources were used. Their localization on the substrate was confirmed by three specific peaks with high signal-to-noise ratios in the Raman spectra. In Figure 5B3 a pixel was assigned for each peak: 1012 cm^−1^ coming from the vibrational mode of phenylalanine is represented by red, 1509 cm^−1^ the ring-breathing mode in DNA bases is yellow, and the Raman mode of tyrosine peak at 1613 cm^−1^ is the blue pixel. The black pixels represent all three peaks and are considered to originate from EVs. To observe the 1613 cm^−1^ peak, the graphene G-peak was subtracted. SEM images confirmed the Raman mapping result of the EVs location on the substrate at three different EVs concentrations. Additionally, the PCA analysis proved that all different EVs detected by this platform clustered into distinguishable groups of EVs isolated from two lung cancer cell lines HCC827 and H1975 with <5% overlap and a sensitivity of >84% (Figure 5B4).

In 2020, Pramanik et al. used a mixed graphene oxide (GO) gold nanostars (GNSs) substrate for ultrasensitive SERS detection of EVs derived from triple-negative breast cancer (TNBC) type MDA-MB-231 cells and HER2(+) type SKBR3 breast cancer cells *via* their specific fingerprint Raman bands [178] (Figure 5C1). Amino functionalized GNSs were covalently linked to GO nanosheets to obtain the mixed-dimensional heterostructure-based GO-GNS substrate. This substrate is capable to highly amplify (∼10^10^) the Raman signal of EVs through a synergistic electromagnetic and chemical enhancement mechanism. The sensitivity of the platform is characterized by LOD of 3.8 × 10^2^ EVs/mL and 4.4 × 10^2^ EVs/mL for TNBC and HER2(+) breast cancer-derived EVs, respectively. The EVs spectra recorded using this hybrid material show that each type of EVs has a specific Raman fingerprint. The lipid bands centered at ∼1605, ∼1260, ∼1056, and ∼970 cm^−1^ are unique for TNBC-derived EVs, while HER2(+) breast cancer cell-derived EVs shows unique protein bands at ∼1634, ∼1198, ∼1101, and ∼1014 cm^−1^ (Figure 5C2). Also, DNA band of EVs isolated from TNBC cells appears at ∼1510 cm^−1^ and DNA bands of HER2(+) breast cancer cell-derived EVs are at ∼1388 cm^−1^ (Figure 5C3).

Also, our group developed an SERS platform for EVs concentration and characterization [179]. The innovative superhydrophobic substrate was able to analyze ultralow sample volumes of EVs isolated from the MC65 neural cell line by concentrating EVs in bowl-like features (Figure 5D1). Specifically, a polystyrene bead-decorated CMOS sensor was used to create a template PDMS replica patterned with nano- and micro-bowls for EVs concentration (Figure 5D2). Next, these voids within PDMS replicas were decorated by AgNPs to generate local electromagnetic field enhancement (hot-spots) and consequently increase EVs Raman signal (Figure 5D3).

Another study employed a similar concept of EVs concentration by developing nanobowl-like plasmonic substrate for EVs capturing and SERS [180]. Specifically, the substrate was prepared by soft lithography on a flexible PDMS substrate using polystyrene beads. Obtained nanobowls were sputtered with a thin silver layer to act as SERS active surfaces (Figure 5E1). Initial SERS measurements are acquired from EVs captured in nanobowls in aqueous solution. Later during spectra acquisition, the water eventually evaporates which may affect the intactness of analyzed EVs which is reflected in the differences in SERS spectra (Figure 5E2). PCA analysis shows that spectra cluster into different groups based on the recording time, as can be seen in Figure 5E3.

Recently, Koster et al. demonstrated the ability of SERS-based analysis combined with machine learning algorithms to detect cancer-derived sEVs despite contamination with lipoproteins [181]. These authors isolated sEVs by known isolation techniques including differential ultracentrifugation, size exclusion chromatography, and density gradient ultracentrifugation. All three methods showed different degrees of lipoprotein contamination, which, however, did not significantly affect the SERS results.

Altogether, these studies validate the use of SERS for label-free characterization of the biomolecular composition of single EVs or EVs in bulk. However, the phenomenon of SERS enhancement by plasmonic resonance depends on the distance between the molecule and nanoplasmonic substrate and decays exponentially with the distance such that it becomes insignificant at distances larger than 10s on nanometers [182]. This limits the applicability of SERS for the characterization of intraluminal content of EVs and makes it more suitable for probing EVs membrane and membrane-bounded molecules.

### 4.3 Surface plasmon resonance spectroscopy

Another label-free sensing method that has been introduced for EVs characterization is surface plasmon resonance (SPR) spectroscopy. SPR is a physical phenomenon that occurs as a result of resonance between the incident light at certain angles of incidence and the collective oscillation of the metal electrons propagating along the metal surface [185]. The SPR measures changes in the refractive index of the material adjacent to the metal substrate surface. An example of such refractive index change is the binding of a macromolecule to the surface of the metal due to immunoreaction. Typical SPR systems include a prism connected to a glass sensor chip coated with a thin film of gold (∼50 nm) (Kretschmann configuration). Biomolecular interactions between an immobilized ligand and analyte from a sample result in changes in refractive index and change in the coupling angle with time. Injection of various concentrations of analyte allows the measurement of association rate constants (*K*_a_) which represents the number of binding events per unit of time and describes ligand-analyte complex formation. Next, is the dissociation phase a change in the coupling angle can be measured in response to the release of the analyte after the sensor surface is washed with buffer. Afterward, the affinity binding constant can be determined using the signal obtained during both phase measurements. This allows SPR sensor to measure concentrations of the analytes including EVs in a sample of interest.

SPR sensors have been applied to detect and characterize biomarkers and hormones in the medical diagnostics field, pathogens and toxins detection for food control purposes, and to detect pollutants in environmental monitoring. In the field of extracellular vesicles characterization SPR has been used to determine EVs concentration [184, 185], biomarker detection [186–189], characterization of surface and intravesicular proteins [190, 191], determination of mechanical properties of EVs [192], and single EV detection [193]. While certainly SPR is a widely used technique in the EVs field, for the purpose of illustrating the potential applications in the clinic, in this review we will specifically focus on articles that describe the use of SPR techniques for the analysis of clinically relevant samples. So far, several SPR biosensors have been developed to detect EVs containing clinically relevant biomarkers. First, the platform termed nPLEX is a nanoplasmonic assay based on periodic nanohole arrays functionalized with antibodies [190]. In this study, EVs were isolated from ascites of 20 ovarian cancer patients and 10 healthy individuals. Detection of EVs was based on the binding kinetics of EVs to CD63, EPCAM, and CD24 antibodies-functionalized nanoholes. The results of the study showed decreased levels of EPCAM and CD24 in patients-derived EVs. Another study reported the development of a nano-plasmon enhanced scattering (nPES) assay for the detection of ephrin type-A receptor 2 (EphA2) protein pancreatic cancer specific EV biomarker [194]. The nPES assay demonstrated the ability to distinguish pancreatic cancer patients (*n* = 49) from pancreatitis patients (*n* = 48) based on a level of EphA2-EVs. Moreover, the platform showed promising results in the staging of tumor progression and monitoring drug therapy response. Next, Liu and co-authors introduces a simple SPR sensing platform for lung cancer diagnosis and used exosomes associated EGFR and programmed death ligand-1 (PD-L1) as biomarkers of disease [195]. The SPR platform detected higher levels of EVs EGFR and PD-L1 in the serum of lung cancer patients compared to healthy individuals isolated exosomes. Most recently, Thakur and co-authors reported the development and application of an SPR-based biosensor for glioblastoma diagnosis [196]. The progression of glioblastoma is characterized by enhanced expression of CD44 enriched EVs. The SPR substrate was fabricated using Titanium nitride nanoholes, and CD44 and CD133 capturing antibodies were immobilized onto the sensor surface. The study utilized EVs isolated from blood and CSF of a glioblastoma mouse model. The biosensor was capable to detect and quantify CD44 enriched EVs with 3.46 × 10^−3^ μg/mL LOD. The described platform supports the potential application of SPR biosensors for EVs-based glioblastoma diagnosis.

Finally, SPR based immunosensors have a great potential as commercially available label-free optical biosensors for EVs characterization due to their high sensitivity and ability to monitor binding events in real-time. However, there are drawbacks of the technique that needs to be addressed. For instance, detection of EVs in complex sample matrices including biological fluids without pre-treatment remains challenging. In addition, SPR sensors may provide false-positive or false-negative results as a consequence of artifactual changes in the refractive index. Also, EVs heterogeneity is rarely addressed in the above-described sensors which may certainly affect the measurements.

### 4.4 Infrared spectroscopy

Fourier-transform infrared (FTIR) spectroscopy is a vibrational spectroscopy technique that primarily uses the ability of chemical bonds within a biomolecule to absorb in the mid-infrared range of the electromagnetic spectrum.

The method describes specific absorption bands of proteins, lipids, and nucleic acids that may be found in the molecular cargo of EVs [197]. Moreover, FTIR provides quantitative spectral data and may highlight possible alterations in EVs biochemical composition based on a clinical condition of a patient. Additionally, FTIR is sensitive to the conformation of the analyzed biomolecules that is not commonly described by conventional EVs characterization techniques and may have valuable clinical information.

From the first study published in 2016 [198], FTIR spectroscopy has shown to be an effective method for EVs characterization. Using FTIR, Mihaly et al., analyzed EVs subpopulations and found differences in protein secondary structure and lipid content among EVs subpopulations [199]. These findings were further confirmed by analyzing EVs subpopulations enriched from cancer cell lines highlighting FTIR as an effective tool for quick EVs subpopulation characterization [200, 201]. Recent studies reported the use of FTIR to characterize changes in the biochemical composition of EVs released from cell cultures mimicking various pathological conditions such as septic shock [202], cancer [201, 203–206], AD [217] as well as the effect of cellular treatment on EV cargo [208]. In addition, IR of single microvesicles by coupling IR and AFM have been reported [209] (Table 2).

### 4.5 Multiphoton microscopy

Multiphoton microscopy (MM) is a powerful technique that allows imaging of cellular and subcellular processes *in vivo*. The method is based on the simultaneous absorption of two or more light photons by the molecule of interest [213]. It had been used to study cell–cell interactions, embryonic development, cancer, and neurology [214]. In the field of EVs research, multiphoton microscopy may offer unique opportunities to explore EVs dynamic (release and uptake) *in vivo*. Specifically, second and third harmonic generation in combination with autofluorescence imaging are suitable for label-free detection and characterization of EVs molecular components including metabolites, structural proteins, and lipids. To our knowledge so far, a single study applied label-free multiphoton microscopy for EVs characterization. You et al. studied EVs isolated from breast cancer tissue using multiphoton microscopy [212]. They were able to visualize EVs, characterize their metabolic profile as well as track EVs release, movement, and uptake. The main finding of the study indicates high enrichment of nicotinamide adenine dinucleotide phosphate (NADPH) in breast cancer patients’ EVs. Finally, the authors reported the ability of the multiphoton microscopy to study EVs’ release, movement, and uptake *in situ*. Indeed, as it is the case for all analytical techniques multiphoton microscopy has its own limitations such as relatively poor spatial resolution, limited penetration depth (250–500 μm), phototoxicity, and costly optical microscopy components.

## 5 Applications

Label-free optical methods have been used to detect and characterize EVs as potential biomarkers for the diagnosis of various diseases including neurological disorders and cancer [215]. Moreover, EVs exhibit great potential to be utilized as drug delivery vehicles for disease treatment. In this section, we will discuss applications of label-free optical methods for the diagnosis of neurodegenerative diseases and different cancer types.

### 5.1 Neurodegenerative diseases

**Figure 6:**
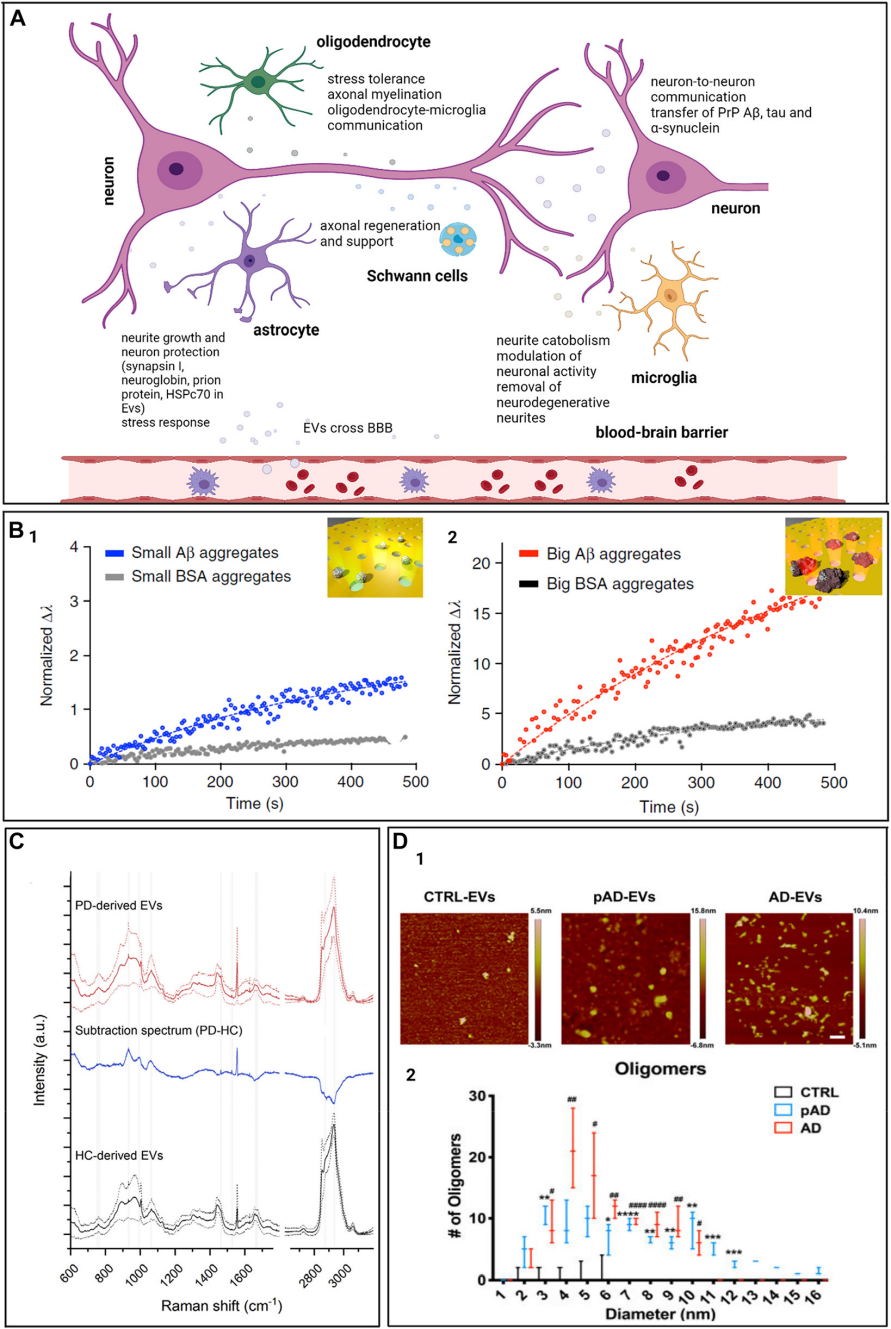
Application of label-free optical methods for characterization of EVs isolated from patients with neurological disorders. (A) Schematic of EVs based intercellular communication in CNS. (B) APEX platform for exosome associated Aβ detection. SPR sensograms showing exosome binding kinetics to (B1) small and (B2) large Aβ aggregates as well as to control bovine serum albumin (BSA) aggregates (adapted with permission from [250]). (C) Raman spectroscopy-based characterization of EVs for detection of patients with PD. Average Raman spectra of PD patients’ serum derived EVs (red spectra) and EVs isolated from the serum of healthy individuals (black spectra). The main spectral differences between the two groups are depicted by blue Raman spectra that is obtained by subtraction between PD-derived EVs Raman spectra and healthy control group EVs Raman spectra (adapted with permission from [248]). (D) Characterization of brain-derived EVs tau oligomers by AFM; (D1) AFM images of EVs associated tau oligomers isolated from AD and prodromal AD (pAD) brains; (D2) graph shows size distribution of Aβ oligomers (adapted with permission from [251]).

The human central nervous system (CNS) is a complex organ where cell-cell communication is crucial for the processing and transmission of information. The main constituents of CNS are neurons and glial cells. The functions of these highly specialized cells are controlled and organized by the communication system of secreted molecules, and EVs have been suggested to play a role in it. EVs are found to be released by all types of CNS cells, including neurons, astrocytes, oligodendrocytes, microglia, and Schwann cells. Existing knowledge indicates the role of glial cells EVs in neuronal maintenance, trophic support, and homeostasis including neurite growth and axonal protection (synapsin I [216], neuroglobin [217], prion protein [218], HSPc70 [219] in EVs) [220–222], myelination [223] and stress response, oligodendrocyte-microglia communication [224–226], and synaptic plasticity [227–230] (Figure 6A). Moreover, EVs are also considered as important mediators of neuronal communication [231] and are able to bypass the blood-brain barrier [232]. In addition to their essential role in the normal physiology of CNS, it is hypothesized that EVs contribute to the pathogenesis of neurodegenerative diseases (ND) *via* the transport of misfolded proteins including prion [233], amyloid-beta (Aβ) [234, 235], tau [236], and *α*-synuclein [237] as well as specific populations of nucleic acids [238]. More importantly, EVs have the potential to be used as readily accessible liquid biomarkers for the detection of neurodegenerative diseases. Nowadays, identification and quantification of potential ND biomarkers require the use of invasive collection techniques, which therefore makes routine diagnosis and monitoring of diseases progression challenging. CSF is considered as a body fluid that reflects molecular processes in the brain. Currently, measurements of Aβ_1-42_, total tau, p-tau, and *α*-synuclein concentration in CSF are used for the diagnosis of some types of neurological diseases. However, invasiveness and the relative high cost of the CSF collection procedure over blood collection are the main drawbacks of the method. In addition, extremely low concentrations of misfolded proteins in CSF and blood of patients limit their use as biomarkers [239]. Therefore, the use of brain-derived EVs carrying ND markers may potentially address aforementioned problems. EVs are found in CSF and blood of patients with various neurological disorders, yet purification of specific brain derived EVs subpopulations remains challenging. Several studies proposed the affinity-immunocapture method to select EVs based on their surface markers. This method has been used to analyze EVs associated biomarkers of Alzheimer’s disease (AD), Parkinson’s disease (PD), and amyotrophic lateral sclerosis (ALS) isolated from patients’ peripheral blood [240–246].

Label-free optical methods have not been widely used for the detection and characterization of CNS-derived EVs. Indeed, NTA and DLS have been used commonly to characterize the size and concentration of EVs, yet these methods did not yield ND specific information through EVs analysis. There are few reports in the literature that described the use of Raman spectroscopy for the identification of ND biomarkers in EVs derived from patients’ blood [247, 248]. Gualerzi et al. demonstrated the use of Raman microspectroscopy to stratify PD patients from a healthy individual group based on their circulating EVs biochemical profile [247] (Figure 6C). The main differences found in spectra of two groups attributed to protein and lipid content of analyzed EVs, where the spectra from healthy control-derived EVs showed higher relative intensities of the amide I protein band and 2800–3000 cm^−1^ lipid band compared to PD EVs spectra. On the other hand, some peaks corresponding to carbohydrates centered at 930, 960, 1370, and 1436 cm^−1^ and lipid peak centered at 1057 cm^−1^ showed higher intensities in spectra of PD derived EVs. Further, the authors applied PCA to discriminate the analyzed groups of patients based on their EVs profile.

Another study used a similar Raman spectroscopy-based approach to identify differences in biochemical content of ALS patients’ EVs [248]. In this study authors isolated small and large EVs from plasma of age-match groups of ALS patients and healthy donors. The main findings indicate a difference in EVs lipid and protein profile of ALS patients compared to healthy patients.

Taken together these studies validated the ability of Raman spectroscopy to identify differences in the EVs biochemical content of PD patients compared to healthy individuals. However, the origin of the described differences in Raman spectra remains to be explored.

Next, there are several reports describing the application of SPR for CNS-derived EVs analysis. Lim and co-authors developed a SPR-based platform termed APEX that was applied to measure exosome-bonded Aβ proteins directly from AD patients’ blood (Figure 6B). Their findings indicated that exosomes preferentially bound to large prefibrillar Aβ aggregates [249] and better reflect neuroimaging results in comparison with total circulating Aβ. Further, the reported results are similar to PET tracers binding behavior that particularly binds to large Aβ aggregates and demonstrate a lower binding activity to smaller aggregates.

Another study utilized SPR to measure the concentration of Aβ, ganglioside M1 (GM1), and translocator protein (TSPO) in AD EVs [251]. In this pilot study, EVs were isolated from the plasma of AD patients (*n* = 10) and healthy individuals (*n* = 10) *via* size exclusion chromatography. Further NTA analysis revealed a higher concentration of EVs in AD patients in plasma (1.18 × 10^11^ particles/ml) compared to healthy subjects (2.34 × 10^10^ particles/ml). Moreover, SPR imaging showed a higher signal of activated microglia EVs in AD patients’ plasma supporting the potential contribution of neuroinflammation to the pathogenesis of AD. Furthermore, SPR enabled simulations detection of potential AD biomarkers including Aβ, TSPO, and GM1. The results of these specific molecules associated with plasma EVs showed a higher amount of all markers of interest in AD EVs compared to healthy controls EVs.

Additionally, AFM has been employed to characterize EVs associated tau oligomers [250]. In this study results of AFM analysis showed the presence of 4–6 nm globular particles, tau oligomers, in fractions of AD and prodromal AD EVs (Figure 6D). In contrast, EVs isolated from control samples did not contain described oligomers. These data suggest enrichment of AD EVs with tau oligomers and their potential pathogenic functions as tau seeding vehicles.

Finally, label-free optical methods have a great potential to be used not only by complementing traditional label-based characterization techniques but to reveal new information about EVs biology and potential application as a liquid biopsy for the diagnosis of ND. For instance, these methods may be used to discover disease-specific conformation of misfolded proteins associated with EVs, where this information is lacking or difficult to explore using current label-based assays.

### 5.2 Cancer

The detection and characterization of EVs as cancer biomarkers is particularly useful for the noninvasive early diagnosis of various types of cancers. Label-free optical methods for EVs analysis can provide valuable information about the EVs structure and molecular content that cannot be achieved using other methods.

**Figure 7:**
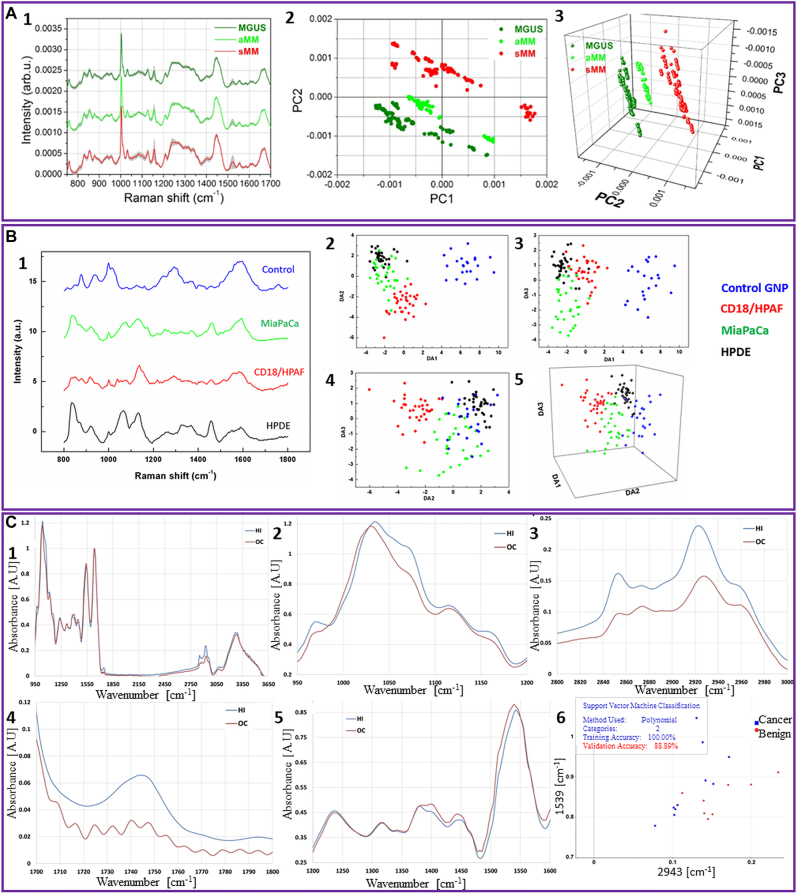
Application of label-free optical methods for characterization of EVs isolated from cancer patients. (A) Raman spectra recorded from EVs isolated from multiple myeloma patients and their PCA analysis; (A1) Average Raman spectra collected from EVs isolated from patients with different stages of multiple myeloma; (A2) PCA analysis shows the stratification of EVs based on the stage of multiple myeloma patients; (A3) 3D scatter plot of PCA analysis (adapted with permission from [254]). (B) SERS spectra of EVs and their PCA and DFA analysis used for the early detection of pancreatic cancer; (B1) SERS spectra of EVs from both normal human pancreatic ductal epithelial cell line (HPDE) and pancreatic cancer cell lines (CD18/HPAF, MiaPaCA); (B2)–(B5) Scatter plots of the discriminant function classifiers DAs showing the capability of the PC-DFA algorithm to differentiate different EVs subpopulations; (B2) Scatter plot of DA1 versus DA2; (B3) Scatter plot of DA1 versus DA3; (B4) Scatter plot of DA2 versus DA3; (B5) Scatter plot of DA1 versus DA2 versus DA3 (reproduced with permission [256]). (C) Label-free FTIR EVs analysis used in the diagnosis of oral cancer. Average IR absorbance spectra of the oral cancer patients and healthy individuals EVs in the range of: (C1) 950–3650 cm^−1^; (C2) 950–1200 cm^−1^; (C3) 1200–1600 cm^−1^; (C4) 1700–1800 cm^−1^; (C5) 2800–3000 cm^−1^; (C6) Support vector machine classification results clearly separate cancer and benign originating EVs (adapted with permission from [205]).

Multiple myeloma is an incurable type of bone marrow cancer formed by malignant plasma cells. Multiple myeloma is always preceded by monoclonal gammopathy of uncertain significance (MGUS), which later slowly progresses to asymptomatic multiple myeloma or symptomatic multiple myeloma. Since the molecular composition of the cargo transported by EVs is dependent on their cells of origin, EVs have been found to be particularly useful in cancer detection and evaluation of the disease progression and in providing clinical info for patient care. Raman spectroscopy is capable of characterizing circulating EVs from a liquid biopsy sample, being a robust technique used not only for cancer detection but also for its prognosis. The spectra of EVs extracted from the serum of multiple myeloma patients in different stages are analyzed by PCA, which effectively stratified these three different clinical conditions [252, 253]. As can be seen in Figure 7A, the EVs’ Raman spectra recorded with an 830 nm laser source are very similar, exhibiting the major known peaks. The most intense peak is phenylalanine at 1003 cm^−1^, amide III, nucleic acids, and fatty acids’ signature are located between 1240 and 1340 cm^−1^, CH deformations in lipids and proteins are at 1440–1450 cm^−1^, while the amide I vibration of proteins band shows up at 1640–1700 cm^−1^ (Figure 7A1). Even though they present similar patterns, they can be clearly separated and stratified by multivariate analysis as shown in Figure 7A2 and 7A3. Although the collected SERS measurements show promising results, poor reproducibility of spectra collection is a major challenge. This is possibly due to the use of SERS substrate with random Au nanostructures. The acquisition of reproducible SERS spectra requires highly ordered SERS substrates, which in turn will increase the cost of the EVs testing due to a more expensive substrate fabrication process. Additionally, SPR biosensors have been used to detect multiple myeloma and stratify multiple myeloma patients from MGUS patients and healthy individuals [254]. These studies reported a 4-fold increase in sEVs concentration in serum of multiple myeloma patients (*n* = 10) compared to healthy subjects (*n* = 10) and MGUS patients (*n* = 5).

Pancreatic cancer is a highly lethal disease characterized by a relentless progression with a five-year survival rate of around 3% due to the late diagnosis when metastasis has already occurred. In an effort to address this issue, Carmicheal et al. proposed an SERS detection platform combined with principal component discriminant function analysis (PC-DFA). In this study, EVs were collected from the supernatant of one healthy and two pancreatic cancer cell lines [255] (Figure 7B1). For detection, positively charged 10 nm AuNPs were used to bind to the surface of isolated EVs and to form a large number of hotspots that highly amplify the Raman signal. As we mentioned before, the EVs surface composition is highly variable between cancer and healthy EVs. The label-free SERS identifies the molecular signature of isolated EVs and the PC-DFA analysis separates them based on spectral differences. To determine the diagnostic accuracy of the method, EVs collected from serum samples of 10 benign control patients and 10 pancreatic cancer patients with early-stage disease were analyzed (Figure 7B2–5). The predictive capabilities still need to be improved, which is not surprising considering the diverse origin of EVs in patient serum, especially in the case of early-stage cancer patients which usually have a large percentage of EVs arising from the normal epithelium. This study demonstrates the potential of SERS combined with PC-DFA analysis for the detection of pancreatic cancer. A recent study by Rasuleva et al. applied FTIR to the study of tumor-derived sEVs as potential diagnostic biomarkers for pancreatic cancer detection [256]. FTIR revealed enrichment of beta-sheet proteins in tumor derived sEVs (*n* = 15) compared to control sEVs (*n* = 15).

Oral cancer is another type of cancer that can be diagnosed using EVs. Oral cancer has a high global incidence with more than 657,000 new cases and 330,000 deaths annually, being the 8th most common cancer in the world [257]. Even if the oral cavity has an easy access for examination, usually this type of cancer is diagnosed in the late stages due to subtle mucosal lesions that appear in the early stages of the disease. By the time of concluding diagnosis, the 5-year survival rate decreases considerably to less than 50%. Therefore, screening and early detection of oral cancer are critical for improving the survival rates and monitoring its progression. A recent study used FTIR in Attenuated Total Reflection (ATR) mode combined with machine learning methods to study and differentiate EVs isolated from the saliva of 21 oral cancer diagnosed patients and 13 healthy individuals [204] (Figure 7C). FTIR spectra highlighted the differences in the EVs content and structure of nucleic acids, lipids, and proteins. These differences can be described mainly by a lower intensity of the oral cancer EVs peaks at 1072 cm^−1^, 2924 cm^−1^, and 2854 cm^−1^ corresponding to nucleic acids and lipids, respectively, compared with healthy EVs spectra (Figure 7C1–5). Also, the relative intensity ratio of peaks centered at 1033 cm^−1^ and 1072 cm^−1^ (I_1033_/I_1072_) and attributed to glycogen/carbohydrates and nucleic acids ratio, is higher in oral cancer-derived EVs spectra compared to control EVs spectra. The PCA–LDA and SVM methods used for the analysis and classification of EVs showed a 100% sensitivity, 89% specificity, and 95% accuracy (Figure 7C6). This clearly proves the potential of this method to be further implemented in clinical facilities for the early diagnosis of oral cancer from liquid biopsy samples.

Finally, using SERS, Ma et al. were able to detect exosomal miRNA-21 as a potential cancer biomarker [259]. The results of the study showed an increased concentration of sEVs miRNA-21 (6.59 × 10^−3^ molecules/EV) in lung cancer patient samples compared to healthy subjects (1.26 × 10^−3^ molecules/EV). Another study reports the application of SERS as a tool for cancer diagnosis [259]. Rojalin et al. used SERS to stratify ovarian cancer and endometrial patients. The authors developed a nanoplasmonic substrate that was chemically pretreated for nonspecific capturing of sEVs. The SERS platform allowed the separation of cancerous sEVs and their differentiation from healthy control derived sEVs. Importantly, this study suggested a critical role of protein corona and glycocalyx in sEVs functions.

Overall, label-free methods for EVs analysis show the potential to differentiate between healthy and cancerous cell-derived EVs, justifying future clinical studies for early diagnosis of various types of cancer using liquid biopsy samples.

### 5.3 Data analysis

Analysis of large data sets is important for providing valuable predictive information. One of the promising, emerging approaches to handle large data sets and discriminate subpopulations of EVs is machine learning (ML). ML algorithms are well equipped to deal with segmentation of large and complex datasets that are typically encountered in heterogenous EVs samples. For example, microscopy images and spectral data from Raman spectroscopy and SERS can be analyzed and processed into various clusters and subpopulations.

ML algorithms can be supervised, meaning that the algorithms are trained on datasets where each training example is explicitly labeled with its corresponding desired output value. This is specifically useful in clinical cases, where diagnostic criteria or biomarkers are known, and the objective is to have a predictive model. Supervised ML models for EVs analysis has been used for the detection of cancers [176, 204, 255, 259] and neurodegenerative diseases [248].

Unsupervised ML algorithms are not trained on prelabeled datasets and therefore must interpret data patterns on their own. Clustering and PCA are examples of unsupervised ML that are used in EVs research [156, 64, 157, 168, 176, 204]. The interesting aspect of unsupervised learning is the potential to discover previously unknown underlying patterns within datasets that the algorithms have identified without human input. For classification problems, an ML model’s performance is evaluated based on the AUC (area under the curve) ROC (receiver operating characteristic) curve. The ROC curve plots the true positive rate (sensitivity) against the false-positive rate for different classification threshold values. The AUC can be calculated as the area under the plotted ROC, and it determines the model’s ability to separate the classes, or the performance of the model through the various classification thresholds. ML models that maximize this metric are very well suited for classification of EVs into healthy or diseased groups. In further studies it is essential to incorporate ML methods to assess different populations of EVs and determine clinically viable diagnostic methods based on optical label-free techniques.

Deep learning (DL) is a subset of ML that uses neural networks to create layers of algorithms to process large-scale datasets. The neural network structure to some extent mimics the interconnection of neurons in the human brain. Predictive classification models have been developed using DL approaches and they can achieve more “human-like” decision-making. EVs research using DL is less common. Shin et al., applied DL approach to classify cancer EVs from healthy patients derived EVs [260]. The DL model was trained using SERS spectra of lung cancer cell lines EVs and human pulmonary alveolar epithelial cell line EVs as healthy control EVs and was able to stratify them with 95% accuracy. Furthermore, the developed DL model was tested using EVs isolated clinical samples. It was able to predict lung cancer for all patients (stages 1A, 1B, and 2B) with an AUC of 0.912 and for stage 1 patients specifically, the model predicted lung cancer with an AUC of 0.910, demonstrating potential for noninvasive, early-stage diagnostics.

## 6 Conclusions and perspectives

As shown in this review, optical label-free methods are currently used as complementary to conventional EVs characterization methods. We anticipate the coexistence of label-free and conventional technologies when the users need to analyze complex analytes such as EVs.

Label-free optical methods enhance the growing field of EVs research by providing effective tools for disease specific biomarkers discovery and simultaneous detection of multiple markers of disease specific molecular fingerprints in small volumes of samples. Among optical label-free technologies described in this review NTA, SPR, and flow cytometry gained the most popularity in the EVs research field. To date, label-free optical technologies have been most successfully applied for the characterization of EVs morphological features such as size, shape, and concentration as well as characterizing the biochemical content of EVs. Furthermore, they have been mostly applied for the detection of EVs-associated markers of various cancer types but have been less commonly used for the diagnosis of other diseases.

Classification of EVs into subpopulations of interest has generally been difficult, due to complex heterogeneity and limited characterization capabilities. Variations between these subpopulations are important to be analyzed to better understand their role in the normal physiology and pathology of the disease. To better quantify these variations, comprehensive and accurate methodologies for the characterization of subpopulations are required. Furthermore, introducing standardized isolation, processing, and analysis methods for different subpopulations of EVs would be a significant benefit to the field, allowing for more consistent and accurate results. In addition, quantitative methods of analysis are critical for establishing EVs as clinical biomarkers and for the development of clinical guidelines.

One of the major current challenges for new technologies to provide accurate diagnosis *via* EVs is the fact that the accuracy of EVs as biomarkers of disease is not always known. It is not always possible to differentiate between the accuracy of a specific biosensing technique and the accuracy of the EVs themselves as biomarkers of pathology. This ambiguity is a key impediment to establishing EVs for clinical use. Biomarkers can be established directly through clinical data or indirectly through a gold-standard methodology. When new biosensors or biosensing methodologies are designed, they are generally developed based on and compared to established biomarkers and their gold-standard detection. In the case of EVs, biosensor development may become problematic, because the accuracy of EVs as biomarkers to diagnose specific diseases is not always established. Consequently, the performance metrics of biosensors tested on unestablished biomarkers is ambiguous, as both the biosensor and the biomarker influence the overall performance.

Within EVs research, ML is an emerging technique used for classifying and clustering samples within large datasets. Various ML algorithms have been employed for classifying clinical samples as well as for investigating underlying patterns within complex, heterogeneous EVs samples to elucidate new subpopulation criteria. Numerous current reports focus on developing predictive models using ML algorithms. Notably, cancers and neurodegenerative diseases have shown to be well classified by predictive models. Integrating ML and including DL into further studies on EVs and their subpopulations or pathology is an important avenue to develop diagnostic criteria, especially combined with high-throughput optical label-free characterization techniques.

Overall, despite many challenges, we expect the field to grow in significance in the near future for liquid biopsy applications, driven by biomarker discovery, standardized sample handling and isolation, technological improvements for characterization, and advanced data analysis methodologies.
